# SARS-CoV-2 population dynamics in immunocompetent individuals in a closed transmission chain shows genomic diversity over the course of infection

**DOI:** 10.1186/s13073-024-01360-1

**Published:** 2024-07-16

**Authors:** Hannah Goldswain, Rebekah Penrice-Randal, I’ah Donovan-Banfield, Craig W. Duffy, Xiaofeng Dong, Nadine Randle, Yan Ryan, Aleksandra M. Rzeszutek, Jack Pilgrim, Emma Keyser, Simon A. Weller, Emma J. Hutley, Catherine Hartley, Tessa Prince, Alistair C. Darby, Niall Aye Maung, Henry Nwume, Julian A. Hiscox, Stevan R. Emmett

**Affiliations:** 1 Institute for Infection, Veterinary and Ecological Sciences, University of Liverpool, Liverpool, L3 5RF UK; 2 Centre for Genomic Research, University of Liverpool, Liverpool, L69 3BX UK; 3 Defence Science Technology Laboratory, Porton Down, Salisbury, SP4 0JQ UK; 4 Centre for Defence Pathology, Royal Centre for Defence Medicine, OCT Centre, Birmingham, B15 2WB UK; 5 British Army, Hunter House, St Omer Barracks, Aldershot, Hampshire GU11 2BG UK; 6 A*STAR Infectious Diseases Laboratories (A*STAR ID Labs), Agency for Science, Technology and Research (A*STAR), Connexis North Tower, 1 Fusionopolis Way, Singapore, #20-10138632 Singapore

**Keywords:** SARS-CoV-2, Transmission cluster, Viral evolution, Minor variants

## Abstract

**Background:**

SARS-CoV-2 remains rapidly evolving, and many biologically important genomic substitutions/indels have characterised novel SARS-CoV-2 lineages, which have emerged during successive global waves of the pandemic. Worldwide genomic sequencing has been able to monitor these waves, track transmission clusters, and examine viral evolution in real time to help inform healthcare policy. One school of thought is that an apparent greater than average divergence in an emerging lineage from contemporary variants may require persistent infection, for example in an immunocompromised host. Due to the nature of the COVID-19 pandemic and sampling, there were few studies that examined the evolutionary trajectory of SARS-CoV-2 in healthy individuals.

**Methods:**

We investigated viral evolutionary trends and participant symptomatology within a cluster of 16 SARS-CoV-2 infected, immunocompetent individuals with no co-morbidities in a closed transmission chain. Longitudinal nasopharyngeal swab sampling allowed characterisation of SARS-CoV-2 intra-host variation over time at both the dominant and minor genomic variant levels through Nimagen-Illumina sequencing.

**Results:**

A change in viral lineage assignment was observed in individual infections; however, there was only one indel and no evidence of recombination over the period of an acute infection. Minor and dominant genomic modifications varied between participants, with some minor genomic modifications increasing in abundance to become the dominant viral sequence during infection.

**Conclusions:**

Data from this cohort of SARS-CoV-2-infected participants demonstrated that long-term persistent infection in an immunocompromised host was not necessarily a prerequisite for generating a greater than average frequency of amino acid substitutions. Amino acid substitutions at both the dominant and minor genomic sequence level were observed in immunocompetent individuals during infection showing that viral lineage changes can occur generating viral diversity.

**Supplementary Information:**

The online version contains supplementary material available at 10.1186/s13073-024-01360-1.

## Background

Coronavirus infectious disease 2019 (COVID-19) is caused by the novel betacoronavirus severe acute respiratory syndrome coronavirus 2 (SARS-CoV-2), which first emerged in Wuhan, China, in 2019 [1]. SARS-CoV-2 resulted from several zoonotic spill over events [2, 3]. As a result, the virus transited through a population bottleneck, with sequencing data indicating that a progenitor virus to SARS-CoV-2 was likely in bats [1]. Subsequent human to human infection has resulted in SARS-CoV-2 genomic diversification from the Wuhan reference sequence. These mutations have arisen through genomic changes through virus and host-mediated single-nucleotide polymorphisms (SNPs), homologous and heterologous recombination events, and insertions and deletions (indels). Host-mediated SNPs can result from cellular proteins, such as APOBEC and ADAR families, interacting with the viral genome. Under selection pressure, these changes may confer a fitness advantage such that resulting genomes can become the dominant viral population, generating novel lineages of SARS-CoV-2. Throughout waves of the pandemic, this has resulted in the dominance of several Variants of Concern (VoCs). Defined by the World Health Organization (WHO), a VoC is a variant that is known to include or have some or all of the following; increased transmission, cause more severe disease, confer immune escape, alter clinical presentation, or decrease the effectiveness of public health measures, diagnostics, vaccines, and treatments. Symptomatology of COVID-19 ranges from asymptomatic, to mild and severe symptoms, through to fatal disease, all of which can present with different profiles depending on the causative variants and VoCs [4–7].

SARS-CoV-2 has a positive sense RNA genome (~ 30 kb) employing a viral and host-derived replication complex, with the catalytic component provided by the viral encoded NSP12 and exonuclease proofreading capacity by the viral encoded NSP14 [8, 9]. Genomic variation has continued throughout the pandemic and continues when the virus entered the endemic phase. This genome divergence has resulted in a wide range of substitutions and indels. In an infected individual, the genome sequence of SARS-CoV-2 is dynamic and sequencing reveals a dominant genomic sequence and minor genomic variants [10–13]. The dominant genome sequence is characterised by the most common nucleotide present at a given position. The first major SNPs diverging from the Wuhan reference genome that conferred increased infectivity and transmission were the D614G substitution in the S protein and the P323L substitution in NSP12, in early stages of the pandemic [14, 15]. The D614G substitution was associated with an increase in transmission and the P323L substitution resulted in viruses with increased replicative advantage [11]. Such selection can occur over a short period of time in one individual, not just between individuals [11, 16, 17]. In addition to dominant genomic variance, minor variation can occur whereby there is a mixed population of nucleotides or amino acids at a given position that occupy less of the proportion of the total than the dominant variant (for example: X at 90% of the proportion and Y at 10%; with multiple amino acids: X 45%, Y 35%, Z 20%).

Minor genomic variants have been hypothesised to transmit between people and this genetic diversity can be observed at different sites of infection within an individual [16, 17]. Identification of viral population genetics can help characterise different evolutionary pressures acting intra- and inter-host alluding to different genetic bottlenecks.

Asymptomatic cases may help propagate transmission and infection throughout populations. This was observed in a care facility where healthcare workers with asymptomatic disease maintained transmission pathways [18]. Human to human transmission is important to understand to assess the dynamics of viral dissemination and curb infection. Transmission studies throughout the COVID-19 pandemic have investigated symptom progression and dynamics. These have shown most notably that transmission was greatest 2 days before and 3 days after an index patient showed symptoms [19] and such studies shaped the policy on non-pharmaceutical interventions [20, 21].

Predominantly, SARS-CoV-2 genomic sequences have been investigated at the dominant genome level, with few studies looking at the dynamics of minor variant transmission. With time, VoCs emerged with examples representing large genomic jumps from circulating strains, as many novel mutations occurred at once, such as with the emergence of Alpha and Omicron VoCs [22]. One hypothesis is that these divergent variants stem from persistent infection in immunocompromised hosts [23, 24].

A potential emerging paradigm, and reflecting previous observations with influenza virus [25], is that in immunocompromised individuals SARS-CoV-2 might be maintained under lower selection pressure than in an immunocompetent individual, across several organs including the lungs, and upper and lower respiratory tract, providing an opportunity for greater genome diversity than virus transmitted between acutely infected individuals [24].

Throughout the pandemic, general sequencing efforts were largely focussed on hospitalised and severely ill patients rather than asymptomatic or mildly ill cases. This was in part due to sample availability and importance around assessing the efficacy of medical countermeasures, although some sequences resulted from samples gathered at nationwide testing sites which were sequenced through many laboratories including COG-UK, including symptomatic and mildly ill cases. However, tying medical records with genomic surveillance has proven difficult/impractical. This resulted in less coverage and/or understanding of the genetics of SARS-CoV-2 in the population that was most responsible for the spread of infection. Such studies, by the very nature of identifying the ‘healthy’ ill, are rare and also include human challenge studies (not all have examined dynamic viral population genetics) [18, 20, 21, 26–31]. Few studies have included an analysis of viral genetics in closed transmission chains [32–34].

This study utilised longitudinal samples from geographically isolated immunocompetent individuals, in peak physical condition (having regular exercise, balanced nutrition and health monitoring) from a single location, to characterise viral evolutionary trajectories in otherwise healthy people. The population genetics of SARS-CoV-2 was characterised in immunocompetent individuals between 20 and 40 years old within a defined transmission chain. Alongside clinical symptomatology this approach was used to determine the number of mutations of the virus between and within individuals and to investigate the scope, diversity, and type of mutational change in immunocompetent patients. Investigation of longitudinal nasopharyngeal swab samples from the closed transmission clusters allowed the tracking of viral evolution throughout the cohort. SARS-CoV-2 sequence differences were identified in the dominant genomic sequence and minor genomic variants with up to 13 dominant substitutions identified over the course of infection in a single participant. The data indicated that between and within individual participants, substitutions could result in a change in lineage of the virus. This implies that transmission of genotypes between individuals can be dependent on time post infection and that immunocompromised hosts are not necessarily required for the generation of variants with larger numbers of SNPs, but may be required for the accumulation of indels.

## Methods

### Participant cohort and sample collection

Nasopharyngeal swabs were collected from 16 SARS-CoV-2-infected participants. Participants lived and worked together in a confined geographical area. During November 2020, there was a SARS-CoV-2 outbreak at this site, participants were tested via RT-qPCR targeted to the E-gene, and positive participants isolated and kept symptom diaries. Sixteen out of 70 individuals onsite present at a spreading event either showed SARS-CoV-2-related symptoms or were tested as part of track and trace efforts to control the outbreak. Subsequently, all individuals were tested for SARS-CoV-2 through E-gene RT-qPCRs. The 16 individuals who tested positive were aged between 21 and 39 and were 1:3 biological female:male; they were immunocompetent and had no comorbidities. Nasopharyngeal swab samples collected for RT-qPCRs were then stored at − 80 °C. Ct values were determined from these. All samples from all participants underwent the same extraction, sequencing, and data analysis as described below.

### RNA extraction and amplification of viral nucleic acids

RNA was extracted from nasopharyngeal swabs at Containment Level 3 using the QIAmp Viral RNA Mini Kit (Qiagen). Samples were then DNase (Turbo DNase, Invitrogen) treated at Containment Level 2 and all further processing completed at Containment Level 2.

### Amplicon library preparation and Illumina sequencing

During library preparation, 8 µl of each RNA sample was converted into cDNA in a reverse transcription reaction using LunaScript™ (Thermofisher), and then amplified by reverse complement (RC)-PCR amplification using the EasySeq™ SARS-CoV-2 Whole Genome Sequencing kit (Nimagen, Netherlands) [35]. The Nimagen kit consists of one PCR-like reaction that acts in two steps to barcode samples and ligate adapters simultaneously using two types of oligo. A universal tail includes a Unique Dual Index (UDI), sequence adapter and universal sequence and also the RC (reverse-complement) primer which contains an extension blocker, universal sequence and the SARS-CoV-2 genomic target sequence reverse complement. In the reaction, the universal sequence and the SARS-CoV-2 target specific primer hybridise to create the SARS-CoV-2-specific RC-PCR primer. This includes the specific SARS-CoV-2 primers with UDI and adapter sequences. The V3 kit used comprises of 154 primer pairs of around 300 bp overlapping the SARS-CoV-2 genome, which are divided into two pools each containing 77 primers. 40 cycles of amplification are used in the PCR. Post-amplification, 1:1 pooling of each amplicon library occurred which was then cleaned using Agencourt AmpureXP beads (Beckman Coulter™, Fisher Scientific, Hampton, New Hampshire). The cleaned amplicon libraries were quantified using a Qubit double-stranded DNA (dsDNA) High Sensitivity Assay kit on a Qubit fluorometer (Life Technologies) and then quality checked on an Agilent 2100 Bioanalyzer (Agilent, Santa Clara, California). The two pools were then combined and denatured. A NovaSeq cartridge (2 × 150 bp run) loaded into a NovaSeq 6000 machine was then used to sequence the denatured amplicon library across a single sequencing run.

Library preparation and NimaGen-Illumina sequencing was conducted as previously described [36]. The sequencing of these 50 samples was conducted across a single sequencing run.

### In silico analysis

Illumina adapters were initially trimmed off raw FASTQ reads using Cutadapt v1.2.1 using the -O 3 parameter to trim any reads which match the adapter sequence with 3 bp or more [37]. Base quality scores were calculated through fastq-stats from EAUtils (https://github.com/ExpressionAnalysis/ea-utils), and all base quality scores for paired reads were between 35.8 and 36.4 (Table S1). Further trimming using Sickle v1.200 was done using a minimum window quality score of 20 (https://github.com/najoshi/sickle); reads shorter than 15 bp after trimming were removed. The trimmed reads were processed through the easyseq_covid19 pipeline v0.9 designed to analyse the NimaGen sequencing data [35]. The pipeline processes were as follows. FASTQ files were trimmed with fastp with default parameters for paired-end data (v0.23.2 https://github.com/OpenGene/fastp [38]). These cleaned reads were mapped to the SARS-CoV-2 reference sequence (NC_045512.1) using bwa mem v0.7.17 [39]. To remove the EasySeq RC-PCR SARS-CoV-2-specific primer pairs, Bamclipper v1.0.0 was used [40]. Lofreq (v2.1.5) was used to call variants with a quality threshold ≥ 20, mutation frequency ≥ 50%, and a depth of ≥ 10 [41]. Bcftools consensus v1.9 [42] was used to generate consensus FASTA sequences.

The consensus FASTA sequences were used to classify lineage and nucleotide substitutions in the viruses. Pangolin (v4.0.6, data model v1.8) was used to designate Pango lineages for samples sequenced with ≥ 85% coverage (N count generated using faCount v377) (https://github.com/cov-lineages/pangolin). Snipit was used to call nucleotide substitutions across the genomes for all samples relative to the Wuhan reference sequence (MN908947.3) using the consensus FASTA sequences as input [43].

The output BAM files generated from the EasySeq pipeline were input into DiversiTools v0.1 (https://github.com/josephhughes/DiversiTools) using the script diversiutils.pl and a custom script to count non-synonymous amino acid variations diversiutils_aa.pl to investigate minor and dominant genomic variation [44]. The -orfs parameter was used to input the coding regions of SARS-CoV-2 to count transitions, transversions, and nucleotide variations per position across proteins. Outputs from diversituils_aa.pl were parsed using a custom parse script to collate non-synonymous and synonymous amino acid counts across the genome [44]. Outputs included variant calls per amino acid position, entropy data per nucleotide position and coverage data. The entropy value called is a measure of uncertainty in the dataset which is used to quantify sequence variability at that site and the entropy file includes the frequency of mutations at individual sites of gene segments. Data was analysed from sequences that had ≥ 85% coverage of at least one base across the genome and subsequently filtered at 20X coverage across the genome (Table S1). Visualisations were generated in R-Studio (v4.2.0) using the following packages for data manipulation: tidyverse v1.3.2, dplyr v1.0.10 and reshape2 v1.4.4. Plots were generated using the ggplot2 package (v3.3.6 https://github.com/tidyverse/ggplot2).

Phylogenetic trees were generated through IQ-TREE (v2.2.2.7) using branch supports with ultrafast bootstrap [45] with the parameters –seqtype DNA -m MFP -B 1000 [46]. The resulting treefile was visualised in iToL (v6) [47].

To analyse sgmRNA counts, LeTRS [48] was used which identifies known and novel leader-TRS sequences from the filtered FASTQ outputs. The LeTRS.pl script was used with the Illumina parameters and the LeTRS_plot.pl script was used to plot normalised and actual counts of sgmRNA. The peak normalised count of leader-TRS junctions with at least 1 primer was plotted from the ‘Known_junction’ output file in R-Studio (v4.2.0) using the following packages: tidyverse v1.3.2, dplyr v1.0.10, reshape2 v1.4.4 and ggplot2 v3.3.6.

To assess whether other related viral species were contaminating results, metagenomic analysis was carried out. Kraken2 v2.1.3 was used with paired end reads parameters with –use-names and –confidence of 0.5 [49]. Bracken v2.9 (https://github.com/jenniferlu717/Bracken) was then run using the same kraken2 taxonomic database to estimate the abundance of species within the samples using the output kraken reports. Krona v2.8.1 (https://github.com/marbl/Krona) was used to visualise species abundances and 100% of sequence reads classified from each sample were from the NCBI taxa severe acute respiratory syndrome-related coronavirus (Taxonomy ID: 694,009) (data not shown). This taxon contains animal coronaviruses, SARS and SARS-CoV-2 suggesting all sequence reads are SARS-CoV-2 as other viruses are highly unlikely. Seasonal human coronaviruses are not included in this taxon and were not reported to have been found in the samples.

## Results

### Transmission and symptoms of SARS-CoV-2 within a defined population cohort

**Fig. 1:**
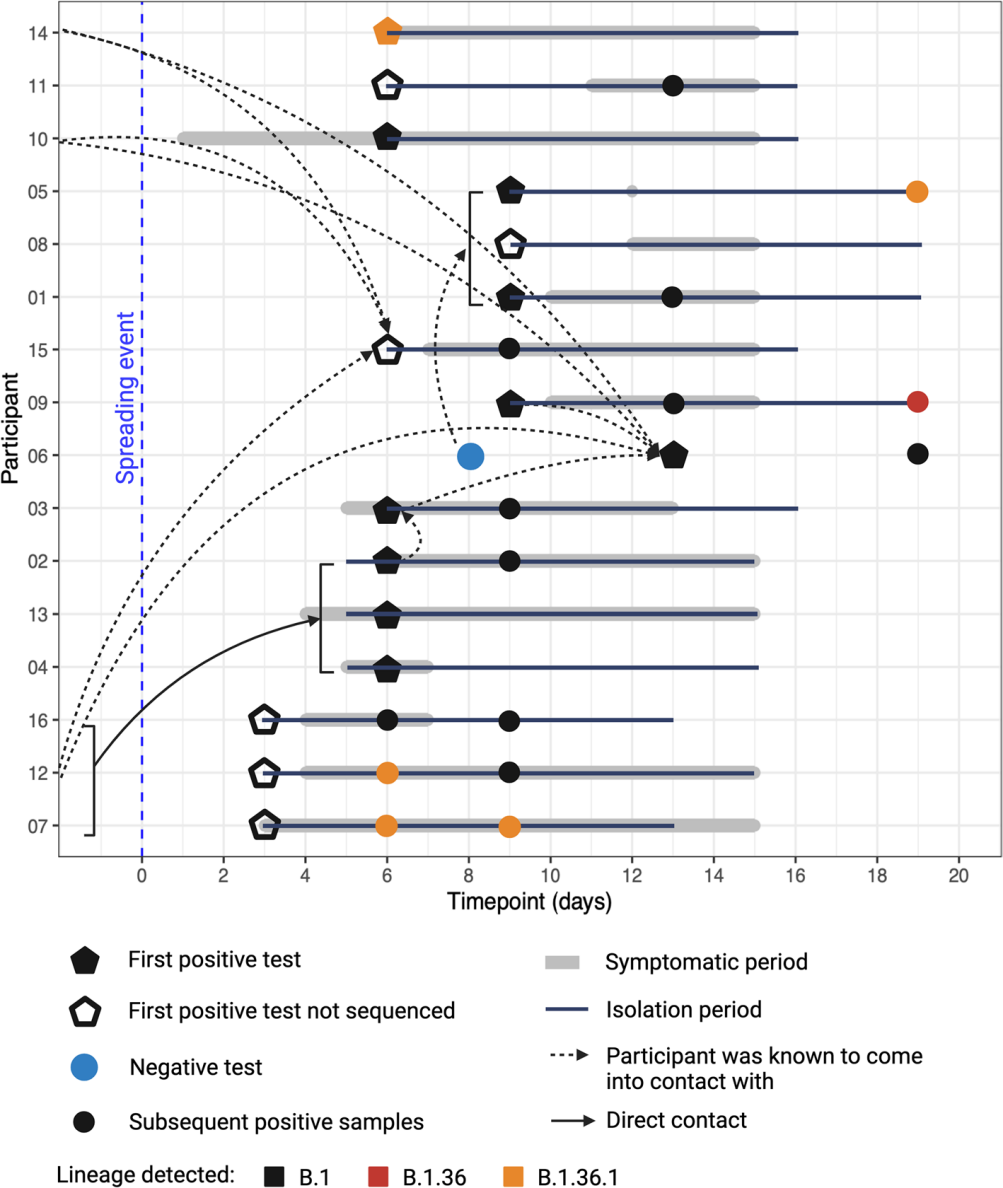
Transmission chain of SARS-CoV-2 between 16 participants (*y* axis) following potential introductions of the virus succeeded by a mass spreading event (at day 0) with 70 people in attendance. The initial positive RT-qPCR test is shown as a pentagon with the only negative test reported shown as a blue circle (Participant 06, who later tested positive). A pentagon with no fill shows a positive test that was not sequenced, and therefore has no associated lineage data (Participant 08 was sequenced but there was not enough sequencing coverage to define a lineage). Symptomatic periods of the individuals are shown in a thick grey line, isolation period in a black line, direct contacts through a solid arrow and participants who came into contact with other participants whilst mixing generally (including living and working together) is shown with a dashed arrow. Subsequent SARS-CoV-2 positive samples from the initial test are shown as smaller circles. Isolation of infected individuals in this cohort only began once population testing had occurred, and those SARS-CoV-2 positive by RT-qPCR were moved to a quarantine location. Participant 06 was the last to test positive, was asymptomatic and was last to isolate during this outbreak recording a negative RT-qPCR test at day 8. Participants in contact at the same time are shown via a bracket, e.g. participants 02, 13 and 04 were all direct contacts of participants 16, 12 and 07. Arrows that begin prior to the spreading event indicate individuals who were in contact with each other on a day-to-day basis i.e. through work and living environment. Lineage information from viral sequences extracted, sequenced and classified using Pangolin are colour-coded: B.1, black; B.1.36, red; B.1.36.1, orange. Note, for consistency, all timepoints are referenced to the mass spreading event and should be considered as days post this event. This figure was created using Biorender

To investigate SARS-CoV-2 population biology during acute infection and transmission, a cluster of cases in a defined population from November 2020, during the pre-vaccination period, was sequenced using a Nimagen-Illumina amplicon-based approach [35] (BioProject PRJNA1012698). Longitudinal nasopharyngeal samples were retrospectively analysed from 16 participants who tested SARS-CoV-2 positive through an E-gene RT-qPCR. These participants were immunocompetent, unvaccinated to SARS-CoV-2, with no known comorbidities or previous (or current at the time) evidence of immune deficiency. The definition of whether an individual is immunocompetent or immunocompromised can be subjective in the absence of defined empirical tests. In this study, we defined our participants as immunocompetent as they had no medication that affected their immune system and had regular health monitoring with no reports of unexpected disease profiles or evidence of non-communicable disease requiring immunotherapy. The participants were 25% biological female and 75% biological male and were aged between 21 and 39 years of age and were living and working as a semi-confined population where potential contact was regularly monitored (Fig. 1).

An initial spreading event is likely to have occurred at which 70 individuals were present where there were two potential introductions of SARS-CoV-2 to this cohort. One introduction was from a SARS-CoV-2-positive individual originating outside our study population and came into direct contact with participants 07, 12 and 16. The second introduction of virus was via another SARS-CoV-2-positive individual external to our study population who had direct contact with Participant 14. Neither of the external cases were present at the spreading event and had no samples collected or sequenced. It is difficult to speculate on the likely lineages of the two viral introductions. Transmission to all participants in contact with these either or both of these two individuals may not necessarily have caused the positive SARS-CoV-2 result and all participants mixed freely at the spreading event, complicating defined transmission routes. At the spreading event, all 70 individuals in this cohort had the freedom to mix. Until day 3 post the spreading event, the study population were mixing as normal in their confined work environment and living spaces. On day 3, the first SARS-CoV-2-positive PCR results were identified and efforts were directed at containment through positive case or contact-tracing isolation (Fig. 1).

**Fig. 2:**
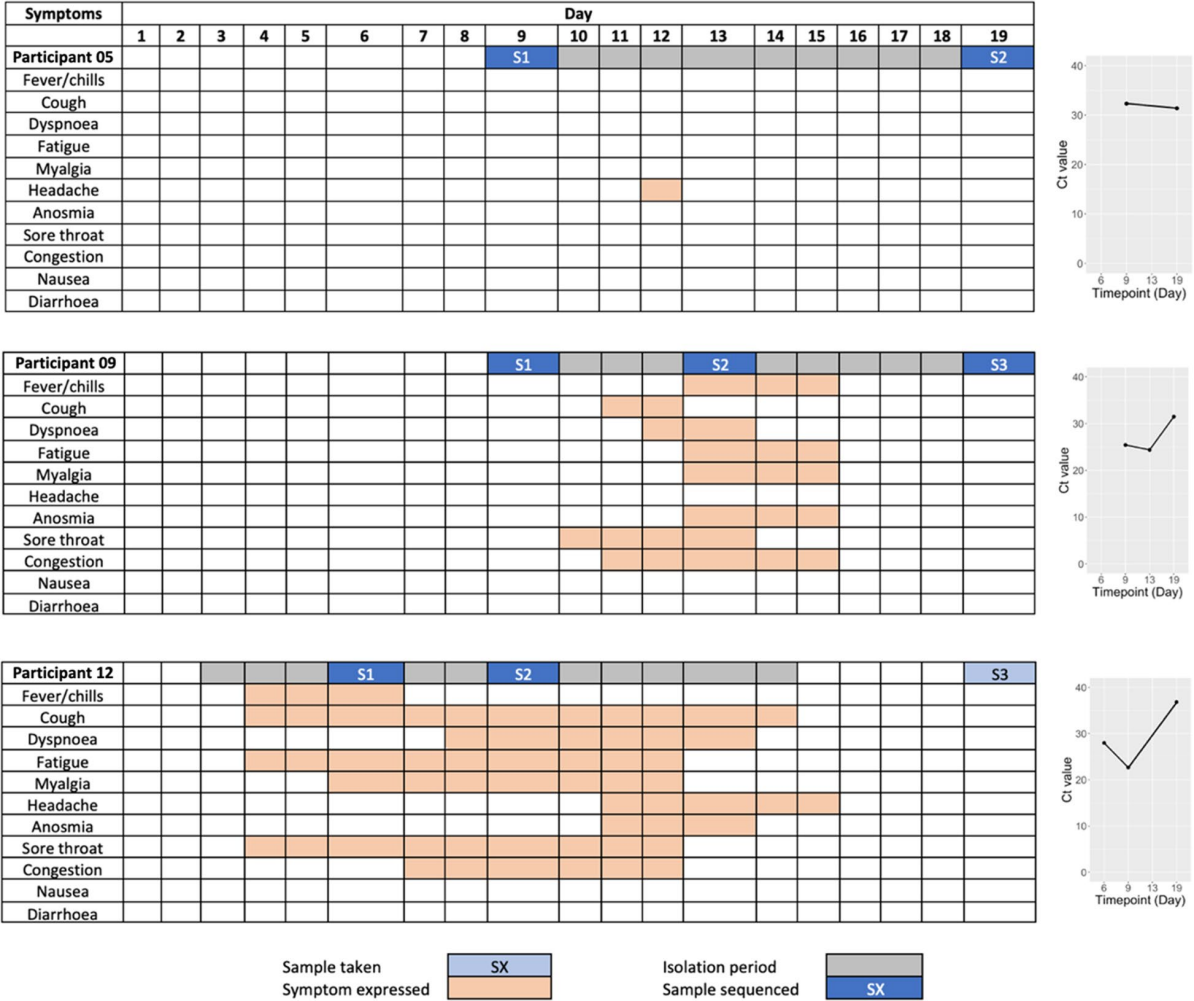
Symptoms presented over time of three exemplar participants, 05, 09 and 12. Sampling took place across 19 days (*x* axis), and sample numbers are denoted as S1-3. All samples were sequenced using the Nimagen-Illumina approach (blue shading: > 85% genome sequence coverage, light blue shading: < 85% genome sequence coverage). Symptoms are shown in orange, and the isolation period per participant is shown in grey. Ct values from an E-gene RT-qPCR are shown on the right over the timepoints sampled. Generally, symptoms experienced were considered mild. Symptom profiles for all participants are shown in Figure S1

None of the infected individuals were defined as immunocompromised or had underlying co-morbidities and were known to be healthy prior to infection. For occupational health surveillance reasons, the cohort and participants underwent close health monitoring and sampling. This monitoring included self-reported health/symptomatology diaries and measurements of viral load (Fig. 2), and where possible, participants were isolated upon a positive RT-qPCR diagnostic result. Symptomatic participants had a range of mild disease; however, the time of onset varied considerably alongside symptom duration (Fig. 2, Figure S1). Generally, symptoms were reported for a short period of 1–4 days, or for a longer period ranging up to and over the 10 days of isolation (Fig. 2, Figure S1). Symptoms included fever/chills, cough, dyspnoea, fatigue, myalgia, headache, anosmia, sore throat, congestion, nausea and diarrhoea, and participants were treated orally with paracetamol. Participant 06 was the only participant to record no symptoms throughout the study. Viral loads were determined using RT-qPCR as a proxy measurement for infectious virus. RT-qPCR of the viral E gene is presented as a cycle threshold (Ct) value in which there is an inverse relationship between Ct and viral load, i.e. the lower the Ct, the greater the viral load.

A positive SARS-CoV-2 RT-qPCR was recorded in seven out of the 16 participants during the pre-symptomatic phase (Fig. 2, Figure S1). Previous studies have demonstrated that viral load peaks on or before symptom onset [50], including the human challenge study [51], which was consistent with profiles seen in this study (Fig. 2). This underlines the capability of SARS-CoV-2 for pre-symptomatic transmission. In Participant 11, the viral load increased during the first two timepoints of sampling to peak at day 13 in sample 3, which was 2 days post symptom onset, suggesting expansion of the viral population from day 9 to day 13 correlated with increased symptoms (Fig. 2, Figure S1). Towards the later timepoints, viral load decreased, and symptoms subsided in most participants. Most symptoms were reported during the middle of isolation and infection periods (Fig. 2). Participant 06 remained asymptomatic throughout infection despite a high viral load which decreased over the two timepoints of sampling (Fig. 2). Participant 06 was the only participant recording no symptoms throughout infection, although Participant 05 only recorded one incidence of symptoms early in infection (an isolated headache on day 12), whilst all other participants presented with a range of symptoms (Fig. 2). Participant 06 underlines the potential for transmission from asymptomatic individuals with high viral loads. The incidence of mild disease in this cohort was reflective of infection in healthy adults in the wider population, where severe disease culminating in fatalities was much more common in the elderly [52] (at least before the advent of vaccination, which this study predates). A correlation was observed between viral load and symptoms, with lower Ct values (between 20 and 30) aligning with more symptoms experienced. Higher Ct values (between 30 and 40) were observed on days where fewer symptoms were reported (Fig. 2, Figure S1).

### SARS-CoV-2 genome sequence was identified in samples from asymptomatic, pre-symptomatic and symptomatic participants

Previous studies using Nimagen-Illumina SARS-CoV-2 sequencing methodologies produced lower-quality outputs in samples with Ct values > 30, reflecting lower coverage [35]. However, in this study, a minimum 85% cut-off was applied to genome sequence coverage, as there were several samples with a Ct value > 30 with sufficient coverage for further analysis. This was to ensure accurate lineage assignment and identification of nucleotide substitutions at both a dominant genome sequence and minor genomic variant level. We note that under this criterion, the highest Ct in which usable sequence was obtained was 32.21 (99.6% coverage), with 4/25 samples having a Ct > 30. From the 16 participants, a total of 50 samples were collected throughout the time course of 19 days and sequenced via an amplicon-based approach. To sequence the 30-kb SARS-CoV-2 genome, overlapping amplicons of around 435 bp were generated as per the Nimagen protocol [35]. Analysis through the EasySeq_covid19 bioinformatics pipeline [35] revealed that out of the 50 samples consensus genomes were generated from 25 samples with > 85% coverage to allow further downstream analysis. Genome coverage plots were generated of all samples sequenced, including those not making the 85% cut-off (Figure S2).

### Three different Pango lineages were assigned in the transmission cluster: B.1, B.1.36 and B.1.36.1

To investigate the dominant genome sequence identified in each participant at different timepoints, the Phylogenetic Assignment of Named Global Outbreak Lineages (Pangolin) tool was used [53]. This assigns the most likely Pango lineage to a SARS-CoV-2 sequence entered into the application based off a dynamic pool of sequences gathered throughout the pandemic [53]. We note that Pango lineage assignment is dynamic and based on both human and machine learning input. The 25 SARS-CoV-2 sequences from these participant samples were analysed through Pangolin [53] (Fig. 2) with a threshold of 85% coverage across the genome. This ensures that when a lineage is assigned, a sub-lineage is not missed through inadequate sequence coverage. Each individual lineage-defining mutation had adequate coverage (> 10X), and therefore was present or absent in the sample rather than it being called due to drop out in sequence coverage.

**Fig. 3:**
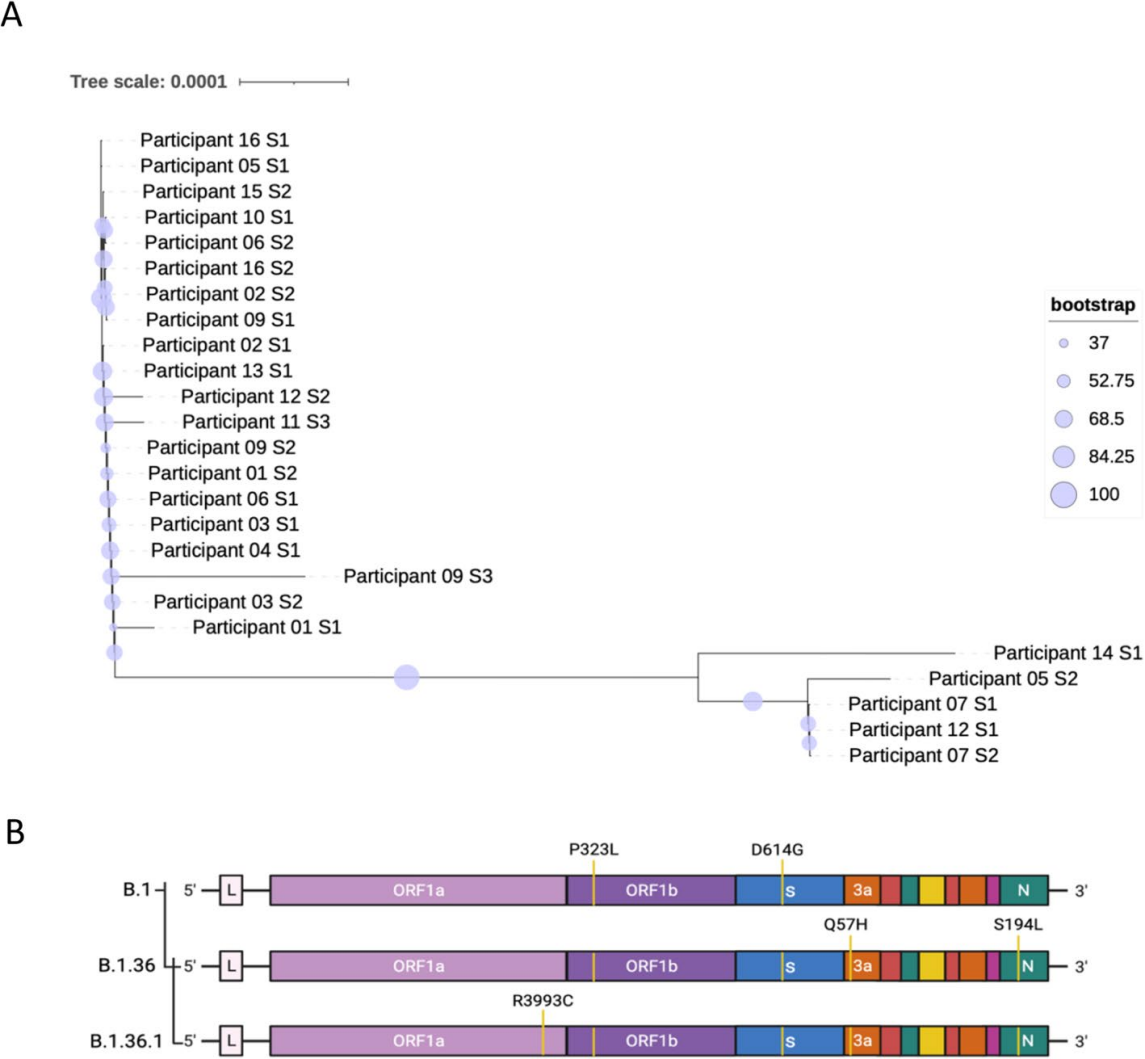
Phylogenetic analysis of the SARS-CoV-2 samples extracted from participant swabs. **A** Unrooted phylogenetic tree showing the relationships between the participant samples of this cohort. Bootstrap values are labelled and shown through purple circles on the branches. Two distinct branches can be seen showing the phylogenetic distance between B.1 and B.1.36.1, with the respective dominant amino acid substitutions across the genome depicted in **B**. Part B in this figure was created using Biorender

Pangolin analysis identified three lineages present in the sample set: B.1, B.1.36 and B.1.36.1 (Table 1). Lineage defining amino acid substitutions for B.1 are D614G in S and P323L in NSP12, and for B.1.36 with the addition of Q57H in ORF3a, S84L in ORF8 and S194L in N and a further R3993C in ORF1a in B.1.36.1 (Fig. 3A). These substitutions mark the divergence of SARS-CoV-2 from the Wuhan reference sequence. All the sequences were classified together into Clade 20A via Nextstrain Clade analysis [54]. This clade represents lineages with the S – D614G mutation present, reflecting the basal pandemic lineage that was globally distributed [54]. Phylogenetic tree analysis revealed two main clusters of viral sequences, separated into B.1, forming the majority of the tree, and B.1.36.1, with the exception being Participant 09 S3 identified as lineage B.1.36 and shown to be more closely related to the B.1 sequences than the B.1.36.1 cluster (Fig. 3B). Sequences assigned to lineage B.1.36.1 formed the smaller cluster. We note that SARS-CoV-2 sequence from participants on different days can be found in different parts of the phylogenetic tree. There was only one sample sequenced from Participant 14 which clustered by itself and was assigned as lineage B.1.36.1, but had additional non-lineage defining features.

**Table 1:** Pango lineage assignments for participants (P) with SARS-CoV-2 samples sequenced with > 85% genome sequence coverage. S1–3 denotes samples 1–3 across time

Participant	Sample	Lineage
01	1	B.1
	2	B.1
02	1	B.1
	2	B.1
03	1	B.1
	2	B.1
04	1	B.1
05	1	B.1
	2	B.1.36.1
06	1	B.1
	2	B.1
07	1	B.1.36.1
	2	B.1.36.1
09	1	B.1
	2	B.1
	3	B.1.36
10	1	B.1
11	3	B.1
12	1	B.1.36.1
	2	B.1
13	1	B.1
14	1	B.1.36.1
15	2	B.1
16	1	B.1
	2	B.1

In all participants aside from 05, 09 and 12 (or those with only a single timepoint sequenced), the Pango lineage classification of the dominant SARS-CoV-2 genome sequence did not change over the course of the disease. Different lineages were observed between separate timepoints in participants 05, 09 and 12 suggesting three possibilities: (i) viral evolution in which the lineage defining mutations occurred during infection, (ii) the presence of a lineage as a minor variant genome at the start of infection that came to dominance during infection, or (iii) infection with a different variant subsequent to the initial infection. These were investigated using the available sequence data and information about the movement of participants.

The evolutionary distance between samples 1 and 2 in Participant 12 reflected differences between viral lineages at timepoint 1 (day 6) and timepoint 2 (day 9) as Pango lineage assignment changed from B.1.36.1 to B.1 in 3 days (Fig. 3). These changes were observed as minor genomic variants at the first timepoint, suggestive of the second possibility above. The lineage assignment of SARS-CoV-2 in Participant 05 changed between timepoints from B.1 to B.1.36.1 occurring within 10 days, from day 9 to day 19 (Figs. 2 and 3A,B). For Participant 09, the dominant genome sequence was lineage B.1 at timepoints 1 (day 9) and 2 (day 13) and was B.1.36 at timepoint 3 (day 19) (Figs. 2 and 3A–B).

Lineage B.1.36 was only identified in a single participant, Participant 09, and only at the third timepoint of sampling. This could be reflective of viral evolution in which the lineage defining substitutions occurred during infection, considering that samples at timepoints 1 and 2 are B.1, thus SNPs occurred in the 6 days between day 13 (timepoint 2) and day 19 (timepoint 3). Aside from Participant 08, with a sample quality too low to sequence, five participants had virus of lineage B.1.36.1 and the other ten B.1 (Table 1).

### Intra-host variation was identified in the transmission cluster

The assignment of SARS-CoV-2 to different Pango lineages from samples both between and during infection in the transmission cluster demonstrated that variation was present at the dominant genomic level. In addition to the lineage defining substitutions for Pango assignment, there were other substitutions from the Wuhan reference sequence (MN908947.3) that were present at the dominant genomic level in the samples. This was investigated using Snipit [43] and applied to sequences with > 85% coverage across the genome (Tables 2 and 3). We note that Pango uses a coverage 50% or greater and therefore we chose 85% to increase confidence in correct calling of substitutions. Figure S3 shows nucleotide mutations across all of the samples sequenced in the cohort, reported through Snipit [43]. The analysis identified additional synonymous and non-synonymous changes that were present in SARS-CoV-2 in all the samples—as well as those substitutions that were used to assign the appropriate Pango lineage. For completeness, starting from the 5′ end of the genome the mutations present in all of the samples were, a C241U synonymous change in the 5′ UTR, followed by changes in the coding region, some of which resulted in an amino acid substitution; NSP3—F106F; NSP12—P323L; NSP14—L280L; S—D614G; ORF3a—Q57H; M—Y71Y and N—S194L (Table 3).

**Table 2:** Amino acid substitutions per virus sequenced with > 85% coverage from participant samples. Substitutions differing within SARS-CoV-2 in participants between timepoints are in bold and substitutions found only once in the sample set are underlined. Sixteen unique substitutions were found, each only in one sample in the cohort. Using default parameters in snipit [43]

Sample	Amino acid mutations	Total mutations
Participant 01 S1 SAMN37347028	5′ UTR C241T; NSP2 A26V, **S32L**; NSP3 F106F, NSP5 M82I; NSP12 L205L, P323L; NSP14 L280L; NSP16 M65I; S D614G, I692I; ORF3a Q57H, L106L; M Y71Y; N S194L, T362I	16
Participant 01 S2 SAMN37347029	5′ UTR C241T; NSP2 A26V, F106F, **S1424F;** NSP5 M82I; NSP12 L205L, P323L; NSP14 L280L NSP16 M65I; S **I231I**, D614G, I692I; ORF3a Q57H, L106L; M Y71Y; N S194L, T362I	17
Participant 02 S1 SAMN37347031	5′ UTR C241T; NSP2 A26V; NSP3 F106F, **S1424F;** NSP5 M82I; NSP12 L205L, P323L; NSP14 L280L; NSP16 M65I; S **V143F,** I231I, D614G, I692I; ORF3a Q57H, L106L; M Y71Y; N S194L, T362I	18
Participant 02 S2 SAMN37347032	5′ UTR C241T; NSP2 A26V; NSP3 F106F; NSP4 M324I; NSP5 M82I; NSP12 L205L, P323L; NSP14 L280L; NSP16 M65I; S I231I, D614G, I692I; ORF3a Q57H, L106L; M Y71Y; N S194L, T362I	17
Participant 03 S1 SAMN37347035	5′ UTR C241T; NSP2 A26V; NSP3 F106F, **S1424F;** NSP5 M82I; NSP12 L205L, P323L; NSP14 L280L; NSP16 M65I; S I231I, D614G, I692I; ORF3a Q57H, L106L; M Y71Y; N S194L, T362I	17
Participant 03 S2 SAMN37347036	5′ UTR C241T; NSP2 A26V; NSP3 F106F; NSP5 M82I; NSP12 L205L, P323L; NSP14 L280L; NSP16 M65I; S I231I, D614G, I692I; ORF3a Q57H, L106L; M Y71Y; N S194L, T362I	16
Participant 04 S1 SAMN37347039	5′ UTR C241T; NSP2 A26V; NSP3 F106F, S1424F; NSP5 M82I; NSP12 L205L, P323L; NSP14 L280L; NSP16 M65I; S I231I, D614G, I692I; ORF3a Q57H, L106L; M Y71Y; N S194L, T362I	17
Participant 05 S1 SAMN37347041	5′ UTR C241T; NSP2 **A26V;** NSP3 F106F, **S1424F**; NSP4 **M324I**; NSP5 **M82I**; NSP12 **L205L**, P323L; NSP14 L280L; NSP16 **M65I;** S **V143F**, **I231I**, D614G, **I692I**; ORF3a Q57H, L106L; M Y71Y; N S194L, **T362I**	19
Participant 05 S2 SAMN37347042	5′ UTR C241T; NSP3 F106F, **A480V;** NSP4 **T189I**; NSP7 **V33V**; NSP8 **R51C;** NSP12, **V410A**, P323L; NSP14 L280L; S **E96D**, D614G; ORF3a Q57H; M Y71Y; N S194L	14
Participant 06 S1 SAMN37347043	5′ UTR C241T; NSP2 A26V; NSP3 F106F, S1424F; NSP5 M82I; NSP12 L205L, P323L; NSP14 L280L; NSP16 M65I; S I231I, D614G, I692I; ORF3a Q57H, L106L; M Y71Y; N S194L, T362I	17
Participant 06 S2 SAMN37347044	5′ UTR C241T; NSP2 A26V; NSP3 F106F, S1424F; NSP4 **M324I;** NSP5 M82I; NSP12 L205L, P323L; NSP14 L280L; NSP16 M65I; S **V143F**, I231I, D614G, I692I; ORF3a Q57H, L106L; M Y71Y; N S194L, T362I	19
Participant 07 S1 SAMN37347045	5′UTR C241T; NSP3 F106F, A480V; NSP4 T189I; NSP6 V84V; NSP7 V33V, D67D; NSP8 R51C; NSP12 P323L; NSP14 L280L; S E96D, D294D, D614G; ORF3a Q57H; M Y71Y; N S194L	16
Participant 07 S2 SAMN37347046	5′ UTR C241T; NSP3 F106F, A480V; NSP4 T189I; NSP6 V84V; NSP7 V33V, D67D; NSP8 R51C; NSP12 P323L; NSP14 L280L; S E96D, D294D, D614G; ORF3a Q57H; M Y71Y; N S194L	16
Participant 09 S1 SAMN37347051	5′ UTR C241T; NSP2 A26V; NSP3 F106F, S1424F; NSP4 **M324I;** NSP5 M82I; NSP12 L205L, P323L; NSP14 L280L; NSP16 M65I; S V143F, I231I, D614G, I692I; ORF3a Q57H, L106L; M Y71Y; N S194L, T362I	19
Participant 09 S2 SAMN37347052	5′ UTR C241T; NSP2 A26V; NSP3 F106F, ***S1424F;*** NSP5 M82I; NSP12 L205L, P323L; NSP14 L280L; NSP16 M65I; S ***V143F,*** I231I, D614G, I692I; ORF3a Q57H, L106L; M Y71Y; N S194L, T362I	18
Participant 09 S3 SAMN37347053	5′ UTR C241T; NSP1 **R124C, L140L**; NSP2 A26V; NSP3 F106F; NSP4 **V180I;** NSP5 M82I; NSP12 L205L, P323L; NSP14 L280L; NSP16 M65I; S I231I, D614G, I692I, **F43F;** ORF3a Q57H, L106L; M Y71Y; ORF8 **F120F;** N S194L, T362I	21
Participant 10 S1 SAMN37347054	5′ UTR C241T; NSP2 A26V; NSP3 F106F, S1424F; NSP4 M324I; NSP5 M82I; NSP12 L205L, P323L; NSP14 L280L; NSP16 M65I; S V143F, I231I, D614G, I692I; ORF3a Q57H, L106L; M Y71Y; N S194L, T362I	19
Participant 11 S3 SAMN37347057	5′ UTR C241T; NSP2 A26V; NSP3 F106F, S1424F; NSP5 M82I; NSP12 **D62Y,** L205L, P323L; NSP14 L280L; NSP16 M65I; S V143F, I231I, D614G, I692I; ORF3a Q57H, L106L; M Y71Y; N S194L, T362I	19
Participant 12 S1 SAMN37347059	5′ UTR C241T; NSP3 F106F, **A480V;** NSP4 **T189I;** NSP6 **V84V**, NSP7 **V33V, D67D;** NSP8 **R51C**; NSP12 P323L; NSP14 L280L; S **E96D**, **D294D**, D614G; ORF3a Q57H; M Y71Y; N S194L	16
Participant 12 S2 SAMN37347060	5′ UTR C241T; NSP2 **A26V**; NSP3 F106F, **S1424F**, **A1736V;** NSP5 M82I; NSP12 L205L, P323L; NSP14 L280L; NSP16 **M65I**; S **V143F**, **I231I**, D614G, **I692I**; ORF3a Q57H, L106L; M Y71Y; N S194L, **T362I**	19
Participant 13 S1 SAMN37347063	5′ UTR C241T; NSP2 A26V; NSP3 F106F, S1424F; NSP5 M82I; NSP12 L205L, P323L; NSP14 L280L; NSP16 M65I; S V143F, I231I, D614G, I692I; ORF3a Q57H, L106L; M Y71Y; N S194L, T362I	18
Participant 14 S1 SAMN37347066	5′ UTR C241T; NSP1 **L88L;** NSP3 **V21V,** F106F; NSP4 **F375F;** NSP6 V84V; NSP7 V33V, D67D; NSP8 R51C; NSP12 P323L; NSP14 L280L, **N518N;** NSP15 **V127F;** S D294D, **T478K,** D614G, **S691S;** ORF3a Q57H; M Y71Y; N S194L	20
Participant 15 S2 SAMN37347071	5′ UTR C241T; NSP2 A26V; NSP3 F106F, S1424F; NSP4 M324I; NSP5 M82I; NSP12 L205L, P323L; NSP14 L280L; NSP16 M65I; S V143F, I231I, D614G, I692I; ORF3a Q57H, L106L; M Y71Y; N S194L, T362I	19
Participant 16 S1 SAMN37347074	5′ UTR C241T; NSP2 A26V; NSP3 F106F, S1424F; NSP4 M324I; NSP5 M82I; NSP12 L205L, P323L; NSP14 L280L; NSP16 M65I; S V143F, I231I, D614G, I692I; ORF3a Q57H, L106L; M Y71Y; N S194L, T362I	19
Participant 16 S2 SAMN37347075	5′ UTR C241T; NSP2 A26V; NSP3 F106F, S1424F; NSP4 M324I; NSP5 M82I; NSP12 L205L, P323L; NSP14 L280L; NSP16 M65I; S V143F, I231I, D614G, I692I; ORF3a Q57H, L106L; M Y71Y; N S194L, T362I	19

**Table 3:** Total proportion of each nucleotide mutation found in viral sequences across the sample set. Overall, 43 different nucleotide mutations were reported across the participant samples over the time course. Using default parameters in snipit [43]. * denotes nucleotide mutations found in < 1% and † in < 5% of SARS-CoV-2 genome sequences submitted to GISAID before March 2024

Nucleotide mutation	Protein	Amino acid mutation	Proportion	Nucleotide mutation	Protein	Amino acid mutation	Proportion
C241T	5′ UTR		1.00	C14408T	NSP12	P323L	1.00
C527T*	NSP1	L88L	0.04	T14669C*	NSP12	V410A	0.04
C635T*	NSP1	R124C	0.04	C18877T*	NSP14	L280L	1.00
C683T*	NSP1	L140L	0.04	C19593T*	NSP14	N518N	0.04
C882T*	NSP2	A26V	0.80	G19999T*	NSP15	V127F	0.04
C900T*	NSP2	S32L	0.04	G20853T*	NSP16	M65I	0.80
G2782T*	NSP3	V21V	0.04	G21850T*	S	E96D	0.16
C3037T	NSP3	F106F	1.00	G21989T*	S	V143F	0.48
C4158T*	NSP3	A480V	0.16	A22255T*	S	I231I	0.76
C6990T*	NSP3	S1424F	0.64	C22444T*	S	D294D	0.16
C7926T†	NSP3	A1736V	0.04	C22995A	S	T478K	0.04
G9092A*	NSP4	V180I	0.04	A23403G	S	D614G	1.00
C9120T*	NSP4	T189I	0.16	C23635T*	S	S691S	0.04
G9526T*	NSP4	M324I	0.32	C23638T*	S	I692I	0.80
C9679T*	NSP4	F375F	0.04	C25521T*	ORF3a	F43F	0.44
G10300T*	NSP5	M82I	0.80	G25563T†	ORF3a	Q57H	1.00
C11224T*	NSP6	V84V	0.16	C25710T*	ORF3a	L106L	0.80
C11941T*	NSP7	V33V	0.20	C26735T*	M	Y71Y	1.00
C12043T*	NSP7	D67D	0.16	C28253T†	ORF8	F120F	0.04
C12242T*	NSP8	R51C	0.20	C28854T*	N	S194L	1.00
G13624T*	NSP12	D62Y	0.04	C29358T*	N	T362I	0.80
G14055T*	NSP12	L205L	0.80				

Apart from substitutions that were common to SARS-CoV-2 in all the samples, there were also changes that were not shared across samples and participants, seen in five samples. These consisted of 16 nucleotide mutations, with most leading to amino acid substitutions. Across the five samples these were as follows: Participant 01 S1 (NSP2—S32L), Participant 05 S2 (NSP12—V410A), Participant 09 S3 (NSP1—R124C and L140L; NSP4—V180I; S—F43F; ORF8—F120F), Participant 12 S2 (NSP3—A1736F) and Participant 14 S1 (NSP1—L88L; NSP3—V21V; NSP4—F375F; NSP14—N518N; NSP15—V127F; S—T478K and S691S) (Table 2). Given that these changes were not shared across all the genomes that were sequenced, and occurred in isolated instances, we postulated that these changes likely represented intra-host viral evolution during infection (Table 2). Aside from the T478K substitution in S (which is a lineage defining mutation for B.1.617.2), the remaining 15 mutations continue to have low global prevalence (https://outbreak.info/). All mutations have been reported previously in the GISAID database; however, 38 out of the 43 mutations were observed in < 5% of global sequences and 35 out of 43 in < 1% of the sequences. Mutations found were screened two-fold: against the ‘Problematic Variants’ list (https://github.com/W-L/ProblematicSites_SARS-CoV2/tree/master), to detect whether mutations were due to sequencing noise, and against the ‘mutational blacklist’ [55] where mutations found are highly conserved thus likely have detrimental effects on viral replication, transmission and survival, so may be a consequence of sequencing artefacts. Mutation T14669C, V410A in NSP12, was present on the blacklist [55] and was found in Participant 05 at timepoint 2 (coverage: 15, V:7; A:8). This mutation does not influence the Pango lineage called and whilst it is on the blacklist, this does not necessarily mean that viruses with these mutations are/were not present in the global population.

Several of the unique mutations were identified in one sample from Participant 14. Only one time point out of the four sampled for this participant contained adequate read depth to analyse the dominant genome sequence for SARS-CoV-2. Although, for the three samples with incomplete coverage, there was adequate sequence read depth in several regions to pull out potential divergence that was maintained from time point 1. These were NSP1—L88L, NSP15—V127F and S—S691S in Sample 2 (Figure S3). These results make it difficult to distinguish between the three possibilities for genomic variation outlined above. Additionally, participants tested SARS-CoV-2 positive at different times throughout the time course (Fig. 1). However, the timing of a positive test in this study did not correlate with increased or decreased genomic variability in the population.

To investigate the prevalence of indels in the samples, the bioinformatic tool LoFreq [41] was used. Across the samples, one indel was characterised in SARS-CoV-2 in sample 1 of Participant 01 starting at nucleotide position 24,010 (amino acid S816 in the fusion peptide region of S) with a deletion of AUUUAUUGAAGAUCUAC replaced with an A. This was observed with a frequency of 0.8 of the viral population with a read depth of 36 at that position with the wild type sequence accounting for the other 0.2. This deletion was not found at the next timepoint, nor were other deletions found across other samples at these positions.

### Analysis of intra-host viral population genetics

**Fig. 4:**
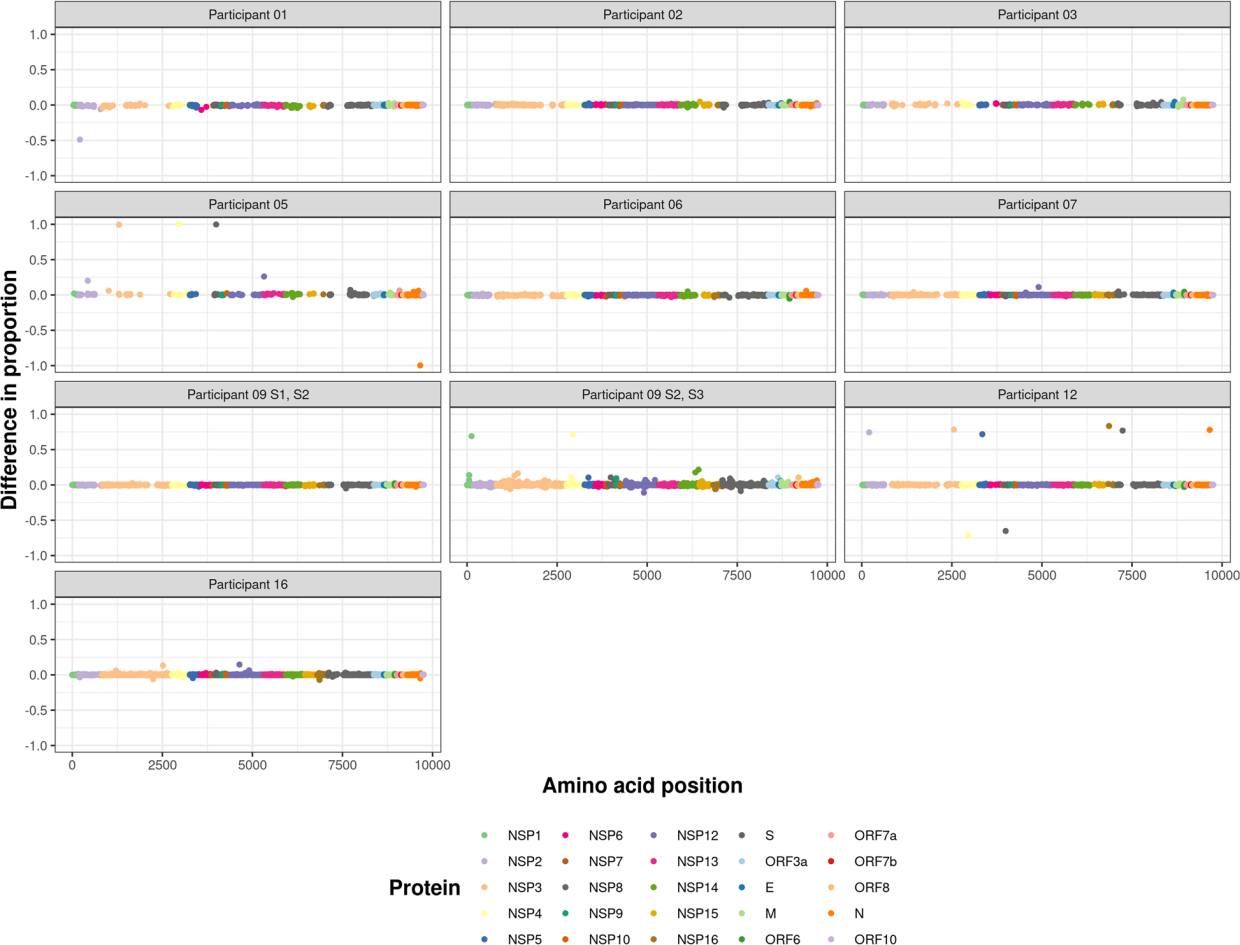
Difference in the proportion of the dominant amino acid at a given position between two timepoints. Most participants had virus that did not have amino acid substitutions differing between timepoints; however, Participants 05 and 12 showed several amino acid substitutions gained (a positive proportion) and several lost (a negative proportion) over time at the dominant level. Participants 01, 07 and 16 showed amino acid substitutions at the minor variant level. Colours denote proteins. Filtered at a coverage of 20X, and minor variants with a coverage of 3X; gaps across the genome reflect coverage below this threshold or variants below this threshold

To further investigate the possible causes of sequence diversity observed in these samples, DiversiTools was used to define the minor genomic variants at an individual level. This tool was used to translate and call the top, second and third most common amino acids and codons at a particular position, hence showing the nucleotide and amino acid substitutions at a given position. Lineage defining mutations occupied a high proportion of the viral population in each participant, showing that they were stable amino acid substitutions from the SARS-CoV-2 reference genome (MN908947.3) (Figure S4). Lower proportions of variation (< 0.5) from the reference genome demonstrated that minor genomic variation was present in samples from these participants (Figure S4). Across timepoints, it was evident that dominant synonymous and non-synonymous amino acid substitutions were maintained at high proportions of the population with little fluctuation (Figure S4, Fig. 4). An exception to this was in samples from Participant 12, where the virus sequenced was classified as two different lineages at different timepoints (Table 1, Figs. 3 and 4). This is illustrated in Fig. 4 through the proportion of dominant synonymous and non-synonymous substitutions being approximately 0.7 of the population, as opposed to a near proportion of 1 in sequences from other samples, suggesting that minor genomic variation accounted for the remaining 0.3.

Minor genomic variation was observed in the samples at low levels, reflective of the error prone nature of coronavirus replication and host cell modification (Figure S4, Fig. 4). Increase in the frequency of minor genomic variants with time could suggest greater fitness was conferred by the particular combination of substitutions, and that with time they may have increased in prevalence to become dominant in the population. Across the samples there were increased levels of minor variation in S, seen where peaks reached a proportion of around 0.15, reflecting potential increased selection of variants or tolerance of variation in this gene. This was also observed in ORF1ab and ORF3a, suggesting that these sites showed greater tolerance for variability compared to the rest of the genome, which may result from increased selection pressure and fitness or the stability of random mutations in those regions (Figure S4).

### Amino acid substitutions from the minor variant population accumulated and became part of the dominant genomic landscape over time in several participants

To investigate dominant and minor genomic variation over time in the sample set, the difference between the proportions of the top amino acids in SARS-CoV-2 was calculated over the timepoints sequenced (Fig. 4). The difference in proportion of the top amino acids between timepoints was minimal, and the majority of amino acids showed less than a proportion of 0.05 change, potentially reflecting a threshold of noise from the sequencing protocol across the genome (Fig. 4). However, some amino acids had a proportion change above 0.1 or below − 0.1, suggesting selection during infection (Fig. 4). To remove any sequencing error that may result in one read skewing the data, the minimum coverage of a minor variant was filtered at a depth of three. Specific examples are discussed.

In samples from Participant 01, in SARS-CoV-2 there was a mixed population of amino acids at position S32 in NSP1 where the minor genomic variant decreased by a proportion of 0.49 from timepoint 1 (day 9) to timepoint 2 (day 13) (Fig. 4). No dominant change to the genome over time was observed; however, a decrease in the minor variant population suggested the dominant amino acid S outcompeted the minor variants present at timepoint 1.

**Fig. 5:**
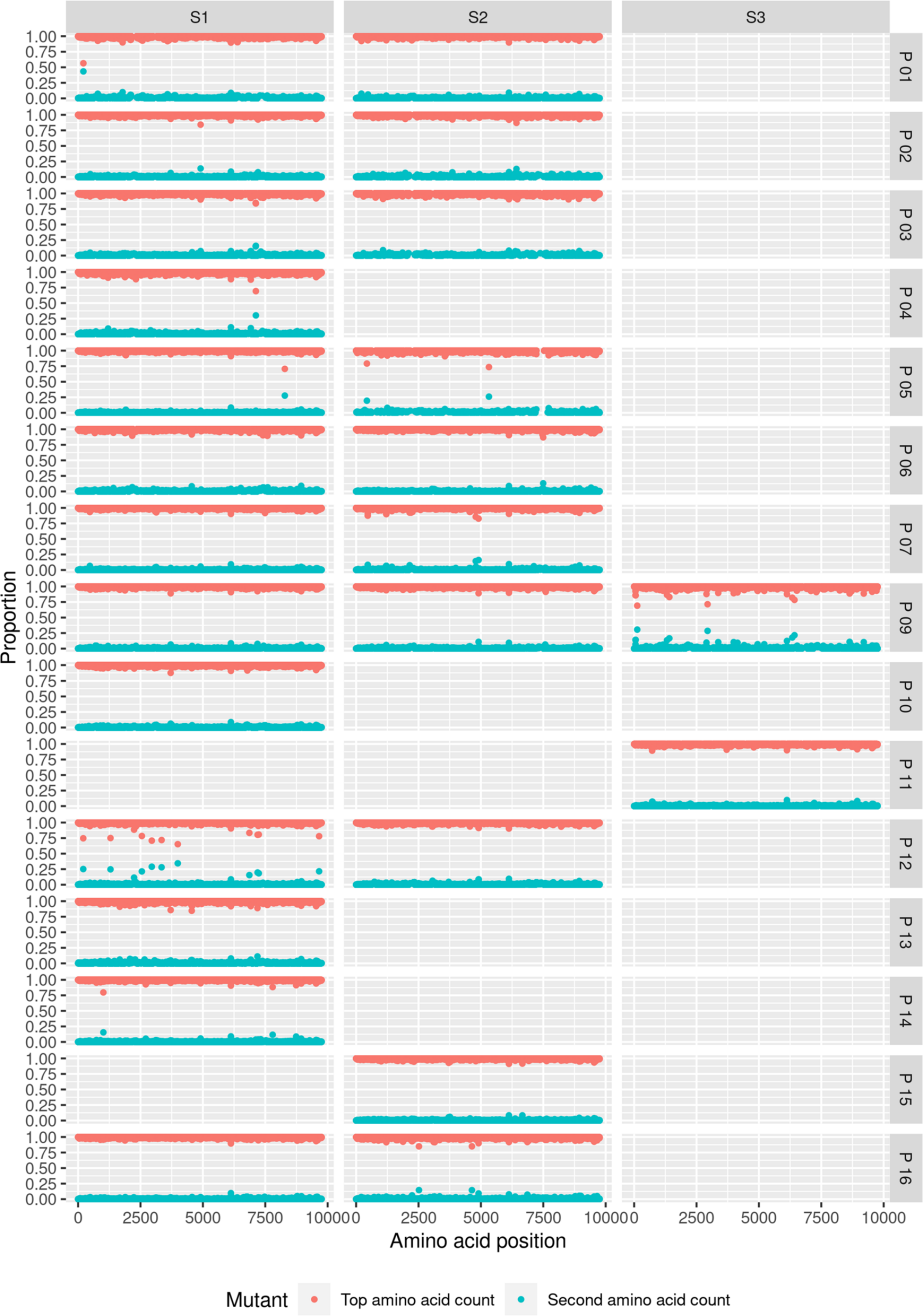
The top (red) and second (blue) most common amino acids across the genome. Many samples had little minor genomic variation (seen with the blue dots); however, at some amino acid positions, there was a mixed population of amino acids shown by the proportion of the second most common amino acid being > 0.1. As timepoints increased minor variation changed seen by changes in the proportion of the top (red dot) and second (blue dot) most common amino acids

In samples from Participant 05, in SARS-CoV-2, three amino acid substitutions were gained over 11 days (NSP3—A480V, NSP4—T189I, NSP8—R51C) and four were lost (NSP2—A26V, NSP5—M82I, N—T362I, NSP16—M65I) compared to the viral genome in the first sequenced sample (Fig. 5). At the minor genomic variant level, at position L922, in NSP12, there was a 0.26 proportion increase, and at I251, in NSP2, a 0.20 proportion increase in non-synonymous substitutions, reflecting the development of a mixed population of amino acids over timepoints 1 and 2 (11 days apart). At position N1187, in S, there was a proportion decrease of 0.28 of non-synonymous substitutions showing that the diversity in amino acid population and minor genomic variation at that position had decreased. These substitutions over the two timepoints in Participant 05 resulted in a change in assignment of Pango lineage from B.1 at timepoint 1 to B.1.36.1 at timepoint 2.

Three longitudinal SARS-CoV-2 samples were sequenced with > 85% coverage across the genome for Participant 09. Very little difference in the minor genomic variation was observed between timepoints 1 and 2 (day 9 and day 13 respectively). However, between the later timepoints 2 and 3 (day 13 and day 19 respectively), minor genomic variation was observed at a frequency above 5%, away from the Wuhan reference genome (Figs. 4 and 5). This suggested increasing viral diversity genome-wide as disease progressed and was not related to viral load in this case (Fig. 2). In particular, at position R124, in NSP1, and position V180, in NSP4, with proportions of non-synonymous changes of 0.69 and 0.71 respectively, amino acid substitutions of R124C and V180I rapidly increased in the viral population outcompeting the amino acids at these positions in the MN908947.3 lineage. At the second timepoint, amino acid C at position 124 in NSP1, was seen as the second most common amino acid, but at a very low level (a proportion of 0.0026), suggesting that it was present and rapidly increased in abundance to outcompete amino acid R at position 124 in NSP1. With substitution V180I, I180 was not observed at earlier timepoints in the minor variant population, only at the dominant level in timepoint 3. Due to increased nucleotide substitutions at timepoint 3 from the previous two timepoints, there was a change in lineage from timepoint 2 to 3 from B.1 to B.1.36, reflecting the cumulative impact of substitutions.

**Fig. 6:**
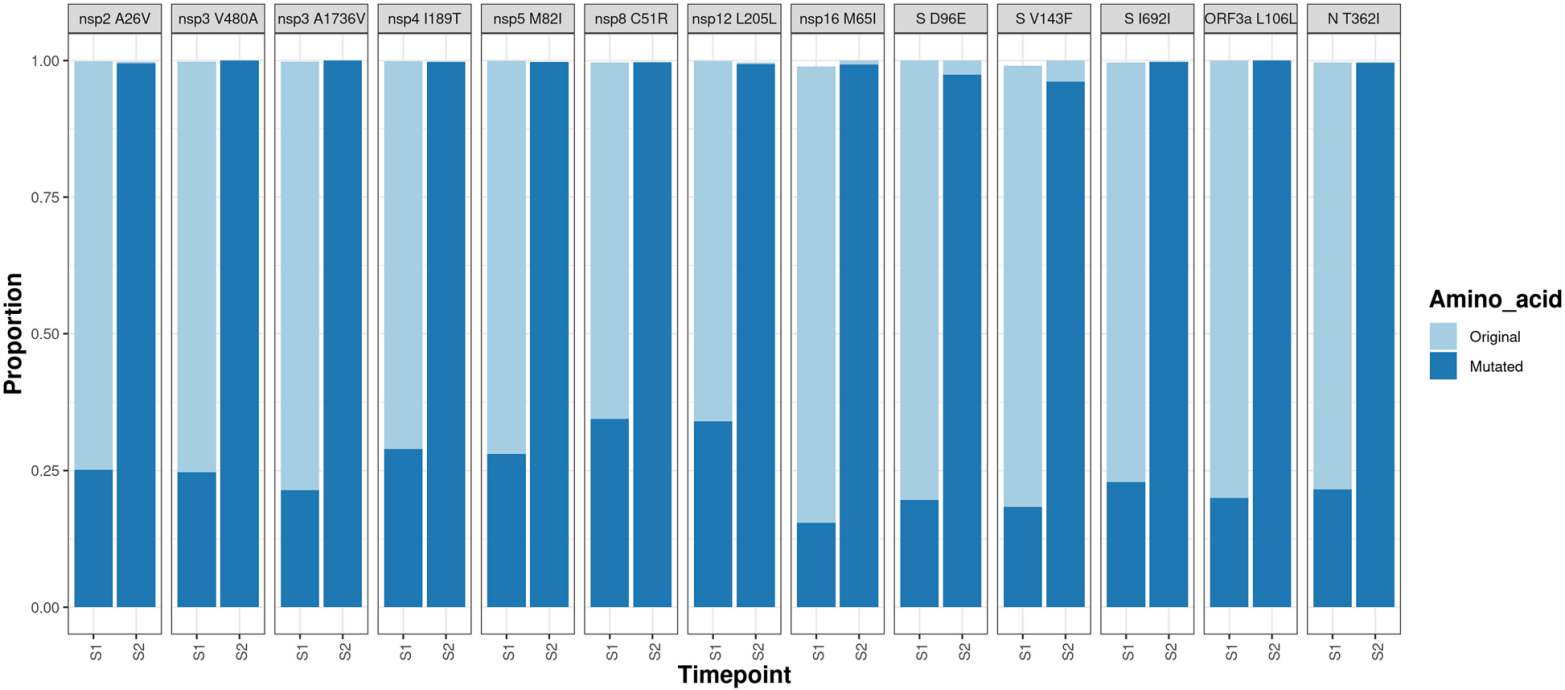
Mixed populations of amino acids were observed at several timepoints (S1 = sample 1 at day 6, S2 = sample 2 at day 9) in Participant 12. In light blue is the dominant amino acid originally, and in dark blue is the amino acid that became dominant over the 3 days between timepoints. From S1 to S2 the amino acid substitution outcompeted the dominant amino acid to become consensus genome sequence

Several of the SARS-CoV-2 samples sequenced sequentially showed elevated variation across the genome compared to other samples, such as in participants 03, 07 and 16 (Figs. 4, 5 and 6). Across these genomes, greater variation was observed at several positions showing higher minor variation levels. There appeared to be more variation across NSP3, NSP12 and S in the proportion of top amino acids, reflecting change over time above other proteins (Figs. 5 and 6). This suggests that these regions were important and conferred increased fitness.

There were low levels of amino acid substitution, which could have reflected the random mutational nature in the genome (Figure S4). These low levels may have also reflected sequencing noise and error generated through the Nimagen-Illumina sequencing. This could be a rare occurrence, due to Illumina NovaSeq instruments having comparatively lower error rates compared to other Illumina technologies with around 0.1% error [56]. LoFreq was used to call variants because it assigns *p*-values to variants to allow for false positive controls, allowing it to predict variants below the sequencing error rate [41]. Additionally, a low background error can be postulated in the sequencing pipeline used due to the selection of a higher read depth and coverage for further analysis. Generally, SARS-CoV-2 in the second longitudinal sample taken had a more diverse minor variant population than from the first sample taken (Fig. 5). This can be seen in Fig. 5 with an increased number of positions across the genome having a proportion of minor variation (blue dots) higher than the corresponding dominant variation (red dots) being lower than in the first timepoint. This suggests that there is potential host modification of the virus to attenuate infection [57] or viral evolution during infection to avoid host defences and confer increased viral fitness [58].

### In SARS-CoV-2 from one participant over time there were a substantial number of mutations suggestive of rapid evolution

Lineage definitions in samples from Participant 12 indicated that the viral sequence classified as different lineages at two successive timepoints: B.1.36.1 at timepoint 1 (day 6) and B.1 at timepoint 2 (day 9). This change in lineage was illustrated through the increase in the minor genomic variation present at day 6 to become part of the dominant genome sequence at day 9 (Fig. 6), potentially illustrative of reversion or the presence of mixed viral populations.

To investigate whether intra-host evolution in the virus occurred over time, the top and second amino acid counts in the sequencing data from the sample set were examined. Specifically, changes in the minor variant population that became dominant in a subsequent sample in an individual were investigated. A mixed population of amino acids was observed at the non-synonymous mutation sites in NSP2—A26; NSP3—V480; NSP4—T189 and M324; NSP5—M82; NSP8—R51; NSP16—M65; and S—E96 and V143, at timepoint 1 (S1, day 6), where the minor genomic variant then increased in the population to become the dominant amino acid at timepoint 2 (S2, day 9, Fig. 6). Several of these substitutions were reversions from substitutions in B.1.36.1 to the reference sequence (MN908947.3) including NSP3—V480A and A1736V; NSP4—I189T; NSP8—C51R and S—D96E (Fig. 6). It seems unlikely for a later lineage to revert to a parent lineage, due to the nature of evolution selecting for fitness advantages. Therefore, it is more likely that Participant 12 had a coinfection of two different lineages at timepoint 1 and one became dominant over time. In this case, that would have contained the reversion substitutions. The other non-synonymous substitutions gained over time instead diverged away from the reference sequence in real time (Fig. 6). The synonymous substitutions NSP12—L205L, S—I692I and ORF3a—L206L were all observed in the samples from Participant 12 with a mixed population of nucleotides at those positions, which resulted in codon changes that became dominant at the second timepoint (Fig. 6). The amino acid substitutions in SARS-CoV-2 between the timepoints reflected mutations accruing over time in the population and outcompeting the predominant amino acid, allowing the minor variant to increase in proportion and thus become the dominant sequence. These amino acid differences between the two timepoints may reflect the presence of a lineage as a minor variant genome at the start of infection that came to dominance during infection. Or this may be characteristic of infection with a different variant subsequent to the initial infection, illustrating a coinfection and virus competition throughout infection. In this small cohort size, the presence of a potential coinfection suggests that these could be common in immunocompetent individuals. This phenomenon may have been prevalent and persisted during the height of the pandemic in populations that allowed close mixing and therefore transmission. This would operate under conditions where no one lineage has a selective advantage and therefore does not dominate the genetic landscape.

### There were higher proportions of non-synonymous than synonymous nucleotide mutations across participants

**Fig. 7:**
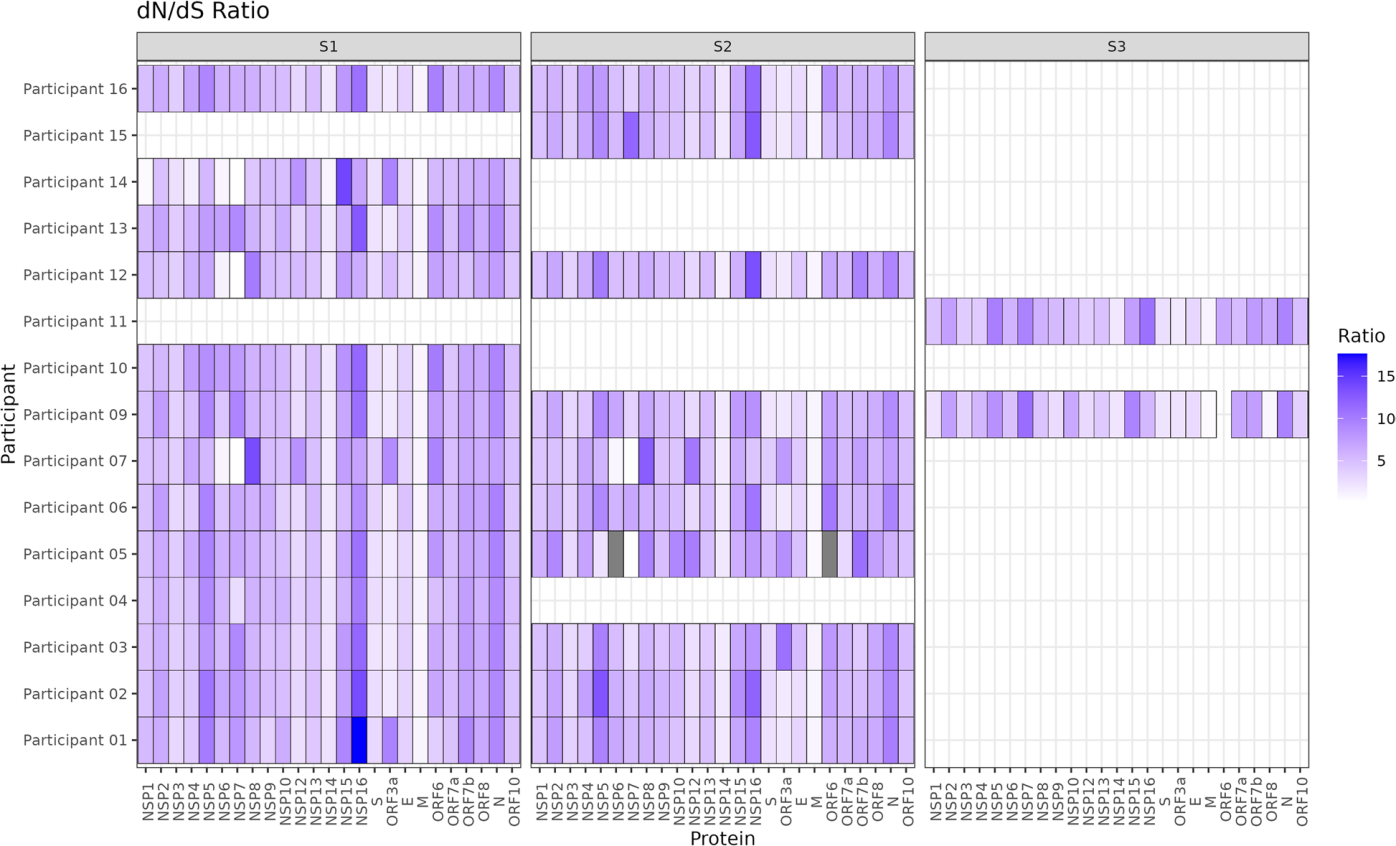
Ratio of non-synonymous to synonymous mutations across proteins in participant samples. S1-S3: Sample 1–3 showing different timepoints. Coverage filtered at 20X. In Participant 05 S2 dark grey represents zero coverage post filtration at 20X in ORF6 and removes an anomalously high ratio of 58.1 in NSP6 which skewed the heatmap scaling. This high ratio is due to very small frequencies of both synonymous and non-synonymous changes being reflected in ratios which would result in a perceived large difference, where, in reality this does not exist

**Fig. 8:**
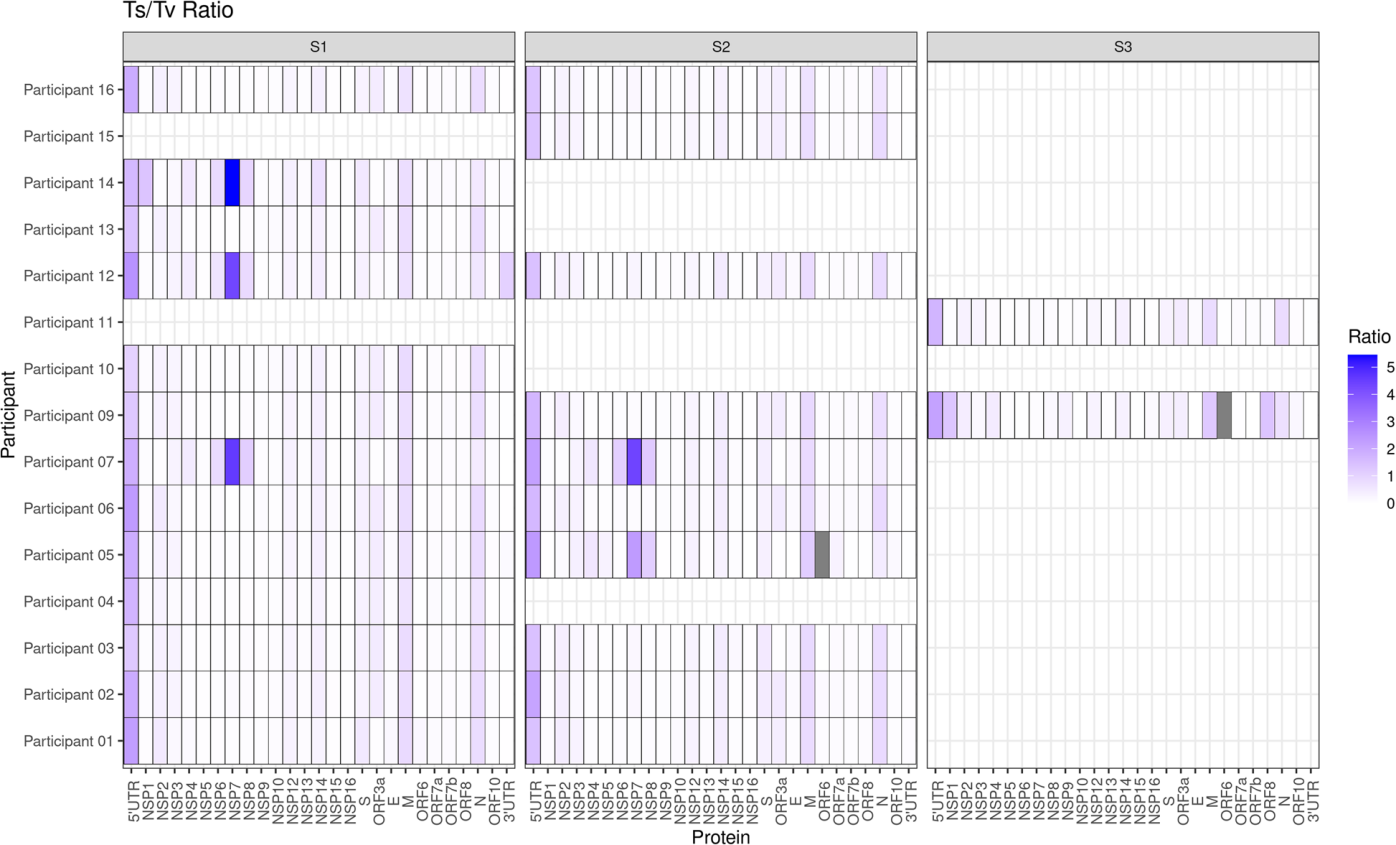
Proportion of nucleotide transitions to transversions across the genome. S1-S3: Sample 1–3 showing different timepoints. Coverage filtered at 20X. The *x* axis shows the position along the SARS-CoV-2 genome and the *y* axis the proportion of transition to transversion (denoted by depth of shading)

Synonymous and non-synonymous mutation counts were also calculated through DiversiTools and revealed increased levels of non-synonymous nucleotide mutations across proteins in the genome compared to synonymous (Fig. 7). Generally, higher proportions of non-synonymous mutations were seen across NSP5, ORF3a, ORF6 and N (Fig. 7). Within the participant sample set, samples with lineages B.1.36.1 showed similar mutational proportion profiles, including similar patterns in both non-synonymous and synonymous substitutions in samples Participant 05 S2, Participant 07 S1 and S2, Participant 12 S1 and Participant 14 S1 (Fig. 7). Proteins with higher proportions of synonymous substitutions included NSP14, S, ORF3a and M, with hotspots in NSP6 and NSP7, and reduced proportions in ORF3a specific to the samples with B.1.36.1 lineages (Figs. 3 and 8). Overall, low levels of 0.5–1% of synonymous and around 1–2% of non-synonymous substitutions per protein were seen throughout the viral sequences (Fig. 7). The high dN/dS ratio observed in Participant 05, S2, NSP6 was due to low frequencies of synonymous and non-synonymous substitutions (around 2–3) skewing the ratio (Fig. 7).

### The proportion of nucleotide transitions and transversions did not change between timepoints but differed between lineages

**Fig. 9:**
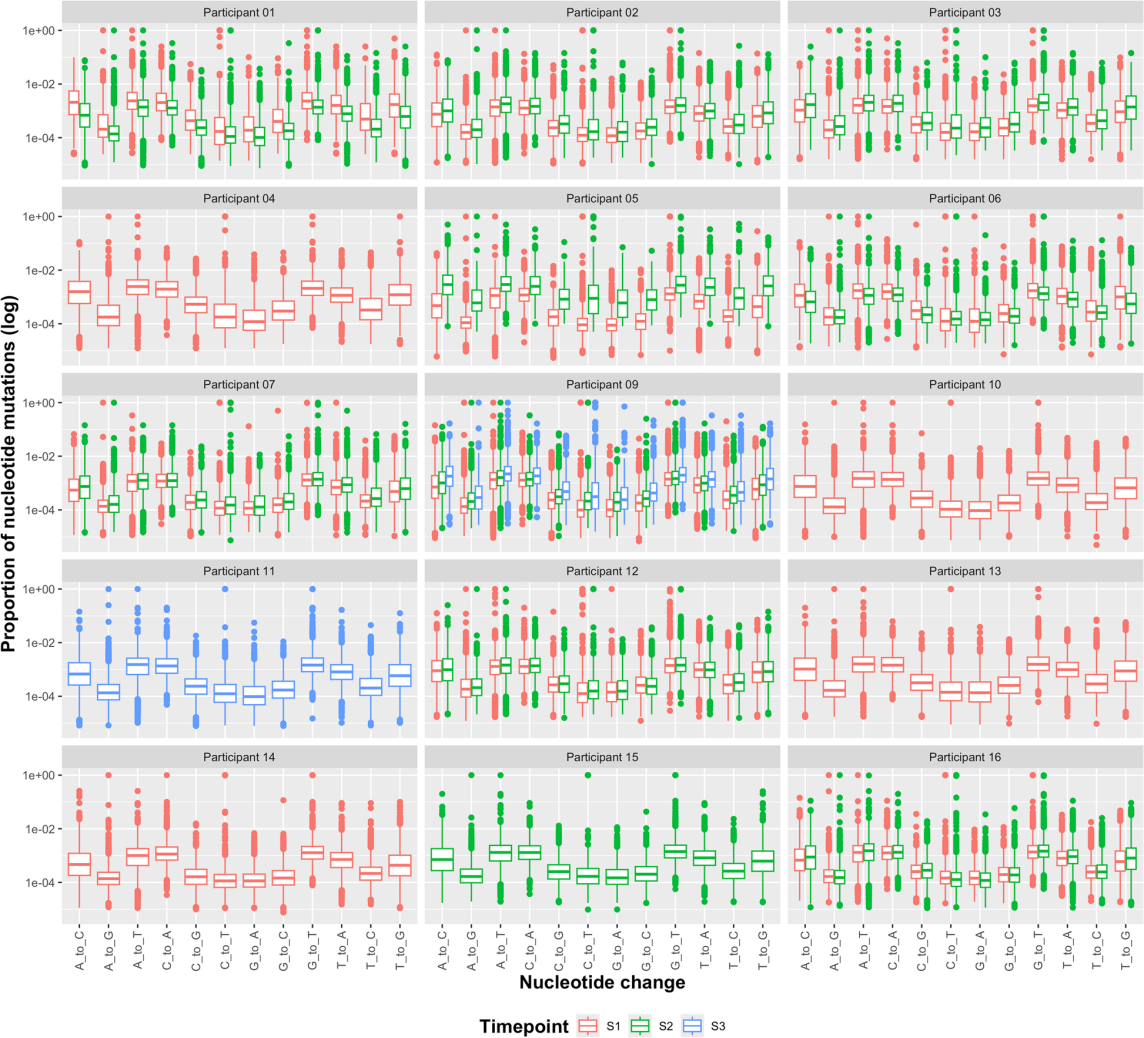
The proportion of base changes across timepoints for participants. Boxplots show the proportion (log scale) of different nucleotide changes (*x*-axis). The different timepoints are shown in different colours (S1 (timepoint 1) in red, S2 (timepoint 2) in green and S3 (timepoint 3) in blue)

More nucleotide transversions than transitions were seen across the genome in most of the samples (Fig. 8). Generally, the proportion of nucleotide transversions remained around 0.003–0.006, compared to nucleotide transitions of a proportion around 0–0.002 (Fig. 9). Hotspots in nucleotide transitions were seen in the 5′ UTR, M and N, with generally low levels (of a proportion between 0 and 0.001) of transitions across the rest of the genome (Fig. 8). Particularly with regard to transition mutations, hotspots were observed with increased proportions across NSP4, NSP6, NSP7 and NSP8 in samples containing virus of lineage B.1.36.1. This was similar to the differences in patterns between lineages seen with non-synonymous and synonymous mutations (Fig. 8). In general, there was no obvious pattern between different timepoints of participant samples regarding the proportion of transition or transversion mutations.

### Hallmarks of reactive oxygen species activity were observed during infection

The most common nucleotide changes seen across SARS-CoV-2 sequenced from the participant samples were A > C, A > U, C > A, G > U, U > A and U > G. Characteristic mutations of host deaminases, APOBEC and ADAR, C > U and A > G respectively [57], were not observed in SARS-CoV-2 in these samples with increasing frequency with time. However, hypothesised hallmarks of reactive oxygen species (ROS) were observed through increased G > U and C > A changes (Fig. 9) [59]. These changes are thought to be associated with ROS, which through oxidising lipids, proteins and nucleic acids may promote mutagenesis of viral genomes and through error catastrophe reduction in infection [59]. The same pattern in terms of distribution and quantity of nucleotide mutations was observed across the participants. Participants 01 and 06 had slightly reduced proportions of the nucleotide changes from timepoint 1 to timepoint 2, whereas the other participants with longitudinal samples showed an increase in the proportion of mutations over time. Participant 09 with three consecutive samples showed consistent increased nucleotide mutations over the timepoints and Participant 05 with high levels of genomic variation between timepoints showed a large increase in mutations over the timepoints.

### Subgenomic mRNA (sgmRNA) was detected in asymptomatic participants showing active transcription in presymptomatic and asymptomatic individuals

**Fig. 10:**
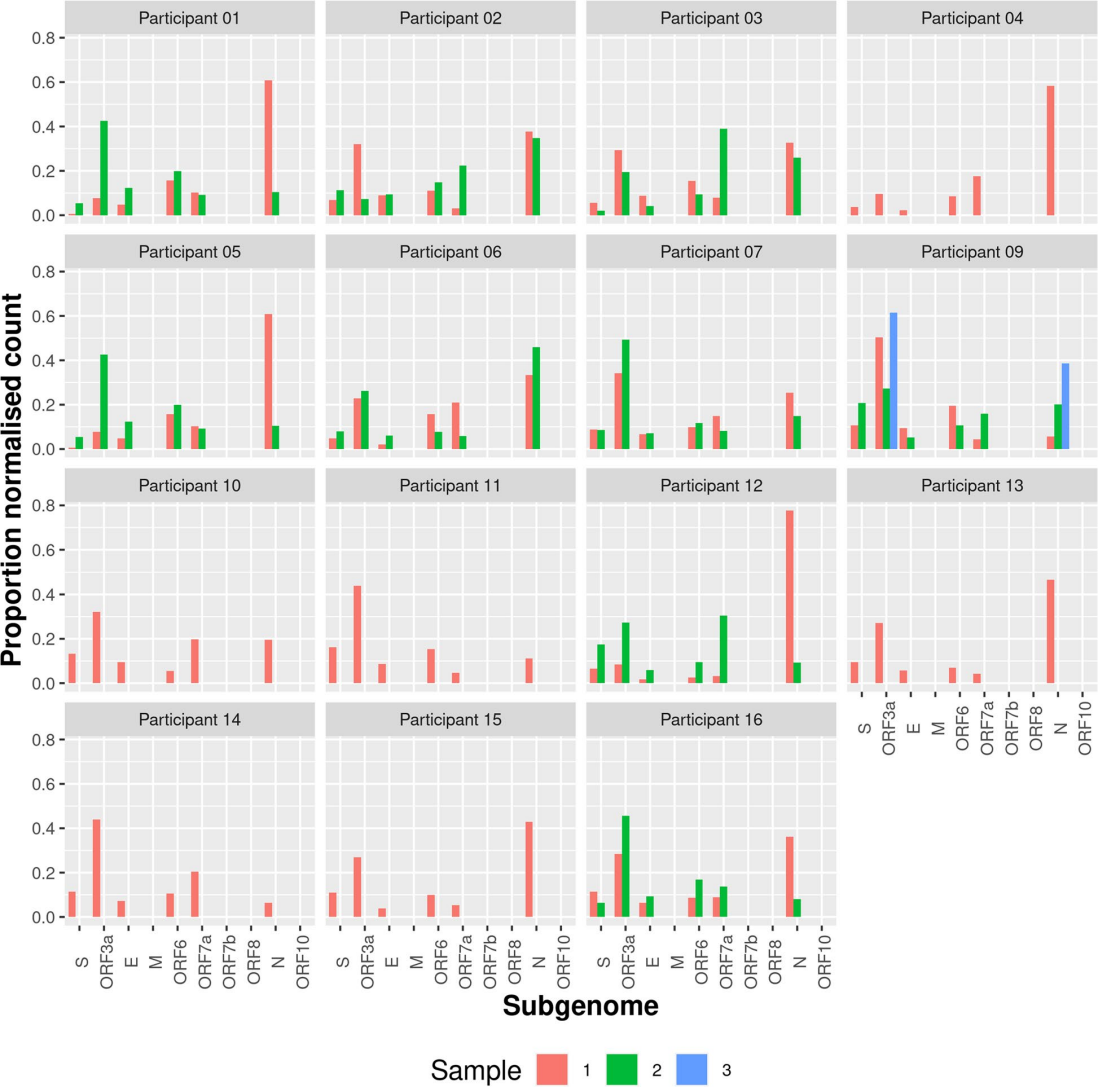
Total proportion of normalised sgmRNA count per participant sample. sgmRNA abundance was more similar intra-host compared to inter-host over time, and sgmRNA quantities varied between participants as well. Generally, there were higher levels of sgmRNA ORF3a and N and very low levels of M, ORF7b, ORF8 and ORF10

The presence of sgmRNA in samples can be evident of active viral replication and transcription in cells as sgmRNA is only synthesised during SARS-CoV-2 infection in cells. Varying levels of different sgmRNAs are seen, with N usually being the most abundant. Unique 5′ sequences called leader-transcription regulatory sequence gene-junctions (leader-TRS junctions) can be identified through sequencing to quantify sgmRNA abundance. LeTRS, a bioinformatic tool developed to identify leader-TRS junctions [48], was used to quantify sgmRNA abundance in this study. Higher levels of N sgmRNA were observed compared to other sgmRNAs in many of the samples, reflecting active viral replication/transcription (or the presence of infected cells in swabs) at time of sampling (Fig. 10). This is also evident in the low Ct values (high viral load) at those timepoints (Fig. 2, Figure S1). This suggested that the peak of infection was between timepoint 1 and timepoint 2. Similar patterns of sgmRNA abundance were seen intra-host over timepoints with many participants showing high levels of ORF3a sgmRNA as well as N (Fig. 10). Lower abundance of sgmRNAs was observed in participants 10, 11, 14 and 15 compared to the rest of the participants, which may be reflective of intra-host fluctuations in viral replication and clearance by the host immune response (Fig. 10).

There was a large variation in the sgmRNA abundance between participants which did not correlate with viral load or symptom presentation. For example, participants 01 and 04 had low sgmRNA abundance, lower viral load comparatively to the cohort and very few symptoms (Figs. 2 and 10, Figure S1). But participants 14 and 15 showed low sgmRNA abundance but varying symptoms and higher viral load (Figs. 2 and 10, Figure S1). sgmRNAs were still detected in asymptomatic infection, indicating that viral replication was active without symptom presentation in some individuals, including participants, 01, 05, 06, 09 (timepoint 1), 11 (timepoint 2) and 16 (timepoint 2) (Figs. 2 and 10, Figure S1).

## Discussion

Genetic variation in SARS-CoV-2 is driven by a combination of SNPs and/or recombination resulting in the insertion or deletion of sequence (indels). The balance between these processes and their contribution to genetic variation is critical in predicting the long-term evolution of SARS-CoV-2. The average number of nucleotide substitutions per year in SARS-CoV-2 is approximately 29.6 (https://nextstrain.org/ncov/gisaid/global/6m?l=clock), which equates to 2.5 per month. From the initial characterisation of SARS-CoV-2 to the end of 2020 (when the samples in this study were taken), the mutational rate was estimated at 22.9 substitutions per year, equating to around 1.9 a month (https://nextstrain.org/ncov/gisaid/global/6m?l=clock). However, sequencing analysis has shown that some variants show higher divergence from progenitor lineages than would be expected given this mutational rate. Partly, this may be down to rapid genomic change through recombination resulting in indels. How this greater than average sequence diversity may arise could be driven by several mechanisms.

Persistent SARS-CoV-2 infection in immunosuppressed individuals has previously been associated with extensive genomic mutations, above the expected mutational frequency [23, 60–63]. Transmission from such individuals has been speculated to be at the root of the evolution of the Alpha and Omicron VoCs [24, 64]. For example, Alpha contained 14 lineage defining amino acid substitutions and three deletions compared to contemporaneous lineages. Omicron BA.1 had 22 additional mutations not seen in combination previously in circulating lineages, including insertions and deletions [5]. Few studies have investigated the genomic variability of SARS-CoV-2 in populations that are immune-competent, ‘healthy’ and non-vaccinated, to provide comparison to data from persistently infected individuals.

In this study, the genomic variability of SARS-CoV-2 was investigated within host and between hosts in a closed transmission chain where longitudinal samples and clinical metadata was used to investigate SARS-CoV-2 population dynamics. The cohort of participants were between 21 and 39 years old with no pre-existing health conditions, comorbidities and no evidence of compromised immunity. Throughout the time course of infection symptoms generally decreased in quantity as Ct values increased (suggestive of viral load decreasing). Viral load peaked around timepoint 1 or 2, early during infection correlating with a peak in symptoms across many of the participants. These trends in symptomology and viral load have been observed in animal models such as ferrets infected with low, medium or high doses of SARS-CoV-2 [65]. Ferrets reportedly presented with more severe symptoms with the higher dose, then medium dose and very few signs of infection with the low-dose inoculations, showing that viral load correlates with observed symptoms in ferret [65] and human infection.

Nasopharyngeal swabs were taken at regular intervals and SARS-CoV-2 identified and sequenced in asymptomatic, pre-symptomatic and symptomatic participants. The predominant driver of genetic change identified in acute infections in ‘healthy’ individuals was SNPs rather than indels. In some cases, SNPs became dominant through a process of minor genomic variants increasing in proportion throughout infection and emerging the dominant genomic sequence (e.g. participants 05, 09 and 12 over a period of less than 11 days (Figs. 6 and 8)). Variation at the dominant and minor genomic level between individuals has been reported in longitudinal studies; however, the substitutions have not resulted in changes in lineage classification [33, 66]. Several of the SNPs observed in this study resulted in changes in viral lineage designation by Pango between timepoints in three out of the 16 participants. Functional analysis of the SNPs reported has not been investigated in this study; however, several of the SNPs carry important functional roles (Table 4). Despite the samples having been collected in November 2020, thereby predating any VoCs, several substitutions observed have been reported in later VoCs, including Alpha (NSP12-P323L, S-V143F), Delta (NSP2-A26V, NSP3-A1736V, NSP4-M324I, NSP12-P323L, S-T478K, S-D614G) and Omicron (NSP12-P323L, NSP15-V127F, S-E96D, S-T478K, S-D614G) (Table 4).

**Table 4:** Functional properties of non-synonymous amino acid substitutions reported in the cohort. Where functional properties have not been investigated in the literature ‘Unknown’ is reported

Mutation	Functional properties
NSP1	
R124C	Destabilisation of NSP1, increasing protein flexibility; may impact immune response or viral replication [67]. Substitutions at R124/K125 have previously been shown to increase destabilisation promoting host RNA decay/reducing host mRNA translation by destabilising binding to 40S ribosomal subunit [68]
NSP2	
A26V	Found in a delta subvariant AY.29 [69]
S32L	Unknown
NSP3	
A480V	Unknown
S1424F	Unknown
A1736V	Prevalent in many delta subvariants [70]
NSP4	
V180I	Unknown
T189I	Unknown
M324I	Found in delta subvariants. Affects the hydrophobic interactions to L321, L323 of the opposite helix; however, effects on stability remain unknown [71]
NSP5	
M82I	Detected as a treatment-emergent mutation (*n* = 3) to Paxlovid in the EPIC-HR trial [72]
NSP8	
R51C	Unknown
NSP12	
D62Y	Unknown
P323L	Located in the interface domain and gives an increased replicative advantage [11]
V410A	Located near the NSP12-NSP7 interface, this mutation has been suggested to lead to alterations in the RNA dependent RNA polymerase activity due to its location in the complex [73]
NSP15	
V127F	Found in Omicron lineages
NSP16	
M65I	Unknown
S	
E96D	Found in Omicron sublineages and emerged in an immuno-compromised patient on day 72 [74]
V143F	In the β9-β10 loop of the NTD, V143 forms rigid interactions so F143 could alter hydrophobicity [75] and be important in antibody recognition. Present in Alpha sublineages
T478K	In the RBD, enhances stabilisation of RBD-ACE2 complex [76] and is found in Delta and Omicron sublineages
D614G	The first Spike mutation reported, found in all lineages and facilitates an open state of Spike, increasing flexibility and cell entry efficiency [77]
ORF3a	
Q57H	Confers an increased dimeric conformation and stability, contributing to the reduced permeability of ions which causes decreased antigenic properties and aids viral evasion of the immune system which could enhance viral pathogenesis overall [78]
N	
S194L	This mutation has been associated with more severe disease [79] and offers a replicative advantage to the virus [80]
T362I	Unknown

The identification of several different lineages in this study may have been due to the de novo generation of a new lineage during intra-host infection or because participants were exposed to two extra-cohort contacts (where no samples were taken but were positive for SARS-CoV-2 subsequently). These individuals may have been infected with different lineages and effectively, co-infection at the same or different time points may have happened. For lineage B.1.36, this was only present at the third timepoint (day 19) of infection in Participant 09, which was the last day of symptoms, infection and isolation. Early samples contained lineage B.1 and the patient was isolated. This suggested that viral diversity resulted from intra-host viral evolution/selection during infection rather than from a coinfection.

One proposed model to account for the increase of minor genomic variants becoming dominant genome sequence in a population is related to the timepoint at which transmission occurs and the frequency of the minor variant in the viral population [11]. Supporting this model is the change in lineage over time observed in participants 05, 09 and 12, as several of the amino acid substitutions that become dominant in later timepoints were distinct populations in the minor variant genomes. This could be due to a narrow transmission bottleneck, allowing variants present in the genomic population to fix during a single transmission event if it allows for increased fitness. Alternatively, a wider transmission bottleneck would allow variants to transmit multiple times in infections and accumulate in frequency more slowly to become fixed. We propose that there were narrow transmission bottlenecks, with minor genomic variants rapidly arising in the population and becoming dominant over timepoints in a single individual.

During infection in one individual, there was a greater than expected frequency of SNPs observed. In Participant 12, there were 13 nucleotide differences in SARS-CoV-2 in samples taken between day 6 and day 9, resulting in 10 non-synonymous and three synonymous amino acid substitutions (Figs. 6 and 7). Interestingly, all of the substitutions were present at approximately 25% of the minor variant population at day 6, and accumulated in the population, outcompeting the previous dominant amino acid, becoming almost 100% of the population at day 9 (Fig. 5). The lineage assignment of SARS-CoV-2 in Participant 12 changed from B.1.36.1 to B.1. This participant may have been infected separately with two different lineages. This paradigm has been established in an example where a patient was co-infected with different SARS-CoV-2 lineages, Alpha and Epsilon [81]. Similarly, data from another case study indicated that two variants were present in an individual patient, potentially as a co-infection. However, one of these variants was more dominant at the start of infection. The patient in the study had a persistent infection and during this time, the lower frequency variant came to dominate [82]. Hence, the drivers of SNP changes in SARS-CoV-2 could be both viral and host. In SARS-CoV-2 identified from Participant 12, the most common nucleotide changes were A > C, A > U, C > A, G > U, U > A and U > G, which increased in frequency from timepoint 1 to timepoint 2 (Fig. 9). Recently, a hypothesis has been advanced to suggest that the G > U and C > A changes observed in SARS-CoV-2 may be attributed to the action of ROS [59].

The data indicated that, in general, the frequency of minor variant genomes in samples was below 5% and remained stable across timepoints. In some individuals this frequency increased with time throughout infection, perhaps because of positive selection pressure. This stability has also been reported across studies including hospitalised patients, individuals in and outside of confined transmission clusters and households [13, 31, 34, 83]. Within the transmission chain analysed here, there were several dominant amino acid substitutions that changed between timepoints, but, in general, a low level of minor variation across the genome was seen. Amino acid substitutions that were characterised once in this cohort were not transmitted between participants, suggesting that these occurred after the spreading event or did not transmit between participants. Minor genomic variation was also not transmitted between participants and did not occur across the same genomic positions in participants. This indicated that viral populations behave randomly as well as responding to selection pressure, causing random mutations to persist and arise in the population.

Participant 14 had virus of lineage B.1.36.1 which was sequenced at timepoint 1 when they had a wide range of symptoms and a high viral load (Fig. 1). Over the 25 samples sequenced, the average number of nucleotide mutations resulting in either synonymous or non-synonymous amino acid substitutions was 17.8, with Participant 14 containing 20, and within that, seven unique amino acid substitutions not seen elsewhere in this cohort (Tables 1 and 2).

Across the participants sequenced, only one indel was characterised in SARS-CoV-2, in sample 1 of Participant 01. This was a deletion located at nucleotide position 24,010, resulting in removal of 17 nucleotides in the S gene, that mapped to the fusion peptide. This deletion was present at a frequency of ~ 80%, with wild type sequence being present with a frequency of ~ 20% as a minor variant genome. However, in a sample from the second time point taken from this participant, only the wild type sequence was identified as the dominant sequence. This suggested that the genomes encoding a defective fusion peptide had been selected against and wild type minor variant genomes supported function—a phenomena reported in Ebola virus population genetics [84].

This study provided a unique opportunity to evaluate a closed transmission system from early events in the COVID-19 pandemic amongst unvaccinated, immunocompetent ‘healthy’ individuals. Although the sample size was only 16 individuals, there are few examples of closed-transmission chain dynamics of SARS-CoV-2 infection in otherwise healthy people. There are limitations to the study, including a relatively narrow age group, the reliance on nasopharyngeal swabs and consideration of a single population due to the reactive nature of the study. Nasopharyngeal swabs were used for ease of sampling and due to the difficulty to obtain ethical approval to conduct bronchiolar lavages on healthy participants without a defined clinical need. Illumina-sequencing analysis of a defined cohort of longitudinal samples, with associated metadata containing symptom and interaction information was used to investigate genomic changes in SARS-CoV-2. The analysis identified both the dominant genome and minor genomic variants in sequential samples. The dominant driver of genetic change was found to be SNPs rather than recombination, and although one deletion was observed, this did not persist as infection progressed. The sequencing analysis showed that a greater than average SNP frequency could occur with 13 nucleotide differences in SARS-CoV-2 reported between sequential samples from the same study participant. This shows that whilst recombination and indels did not appear to occur in high frequencies, the major genomic changes in ‘healthy’ individuals were associated with SNPs.

## Conclusions

Investigation of SARS-CoV-2 infection in immunocompetent individuals has been limited throughout the pandemic. The sequences analysed in this study from SARS-CoV-2 are from healthy participants infected with SARS-CoV-2 and sampled longitudinally, allowing viral evolution to be characterised throughout infection. In several participants, sequence changes over time resulted in lineage classification changes. Initially, several of these changes were observed as minor genomic variants, which then accumulated to become dominant genomic sequence over time and one participant had a potential coinfection. These are both examples of how lineages can evolve in immunocompetent populations and increase the viral diversity in populations throughout the pandemic and as the virus moves towards endemicity.

### Supplementary Information

Additional file 1: Figure S1. Participant symptomatology during the study with corresponding Ct values from positive E-gene RT-qPCR results. Symptomatology reported over the course of the study from day 0 (the timepoint of the super-spreading event) showing when the samples were taken (light blue shading) and their corresponding Ct values from a positive E-gene RT-qPCR test (on the right-hand graph). Symptoms reported (shown down the left-hand side) are shown in light orange, isolation period is shown in grey and samples that were sequenced subsequent to collection are shown in dark blue. Figure S2. Genome sequencing coverage and depth plots for all SARS-CoV-2 samples collected from participants (timepoints are shown as S1-4 where S=sample as timepoints were different between participants), including those with <85% coverage which were not subsequently analysed. Depth was calculated using SAMtools to give coverage across the length of the genome and depth per nucleotide position. Participants (P) are down the right-hand side, with timepoints shown across the top and genome depth using free axes on the y-axis. Figure S3. Nucleotide mutations across the genomes of all participant samples compared to the reference genome (MN908947.3). Diagram of all the nucleotide mutations where sufficient coverage was obtained (20X at that position), insufficient coverage is reported as N at that position generated via snipit [43]. Figure S4. Proportion of non-synonymous (blue) and synonymous (orange) amino acid variation across the genomes of the different participant samples (S1, S2, S3) with >85% coverage, compared to the reference genome. Dominant amino acid substitutions were observed at a proportion of >0.5, with many lineage defining mutations near a proportion of 1, and minor variants can be seen at a proportion of generally <0.5 and generally at a low level across the genome. Coverage filtered at 20X. Table S1. Coverage of samples sequenced when filtered at 85% coverage and then 20X and 10X depth and base quality scores of reads post trimming.

## Data Availability

The dataset supporting the conclusions of this article is available in the Sequence Read Archive (SRA) repository under the BioProject PRJNA1012698 (https://www.ncbi.nlm.nih.gov/bioproject/?term=PRJNA1012698): Longitudinal sequencing of SARS-CoV-2 in immunocompetent individuals raw sequence reads [85]. Custom code used in this study is available at the github repository: [44].

## Abbreviations

SARS-CoV-2: Severe acute respiratory syndrome coronavirus 2
COVID-19: Coronavirus Disease 2019
SNP: Single-nucleotide polymorphism
VoC: Variant of Concern
WHO: World Health Organization
ROS: Reactive Oxygen Species
sgmRNA: Subgenomic mRNA
