# Supplementary material for: SARS-CoV-2 population dynamics in immunocompetent individuals in a closed transmission chain shows genomic diversity over the course of infection
Source: Genome Med. 2024 Jul 16;16:89. doi: 10.1186/s13073-024-01360-1 (PMC11251137; doi:10.1186/s13073-024-01360-1)
Supplement: Supplementary file 1 — Additional file 1: Figure S1. Participant symptomatology during the study with corresponding Ct values from positive E-gene RT-qPCR results. Symptomatology reported over the course of the study from day 0 (the timepoint of the super-spreading event) showing when the samples were taken (light blue shading) and their corresponding Ct values from a positive E-gene RT-qPCR test (on the right-hand graph). Symptoms reported (shown down the left-hand side) are shown in light orange, isolation period is shown in grey and samples that were sequenced subsequent to collection are shown in dark blue. Figure S2. Genome sequencing coverage and depth plots for all SARS-CoV-2 samples collected from participants (timepoints are shown as S1-4 where S=sample as timepoints were different between participants), including those with <85% coverage which were not subsequently analysed. Depth was calculated using SAMtools to give coverage across the length of the genome and depth per nucleotide position. Participants (P) are down the right-hand side, with timepoints shown across the top and genome depth using free axes on the y-axis. Figure S3. Nucleotide mutations across the genomes of all participant samples compared to the reference genome (MN908947.3). Diagram of all the nucleotide mutations where sufficient coverage was obtained (20X at that position), insufficient coverage is reported as N at that position generated via snipit [43]. Figure S4. Proportion of non-synonymous (blue) and synonymous (orange) amino acid variation across the genomes of the different participant samples (S1, S2, S3) with >85% coverage, compared to the reference genome. Dominant amino acid substitutions were observed at a proportion of >0.5, with many lineage defining mutations near a proportion of 1, and minor variants can be seen at a proportion of generally <0.5 and generally at a low level across the genome. Coverage filtered at 20X. Table S1. Coverage of samples sequenced when filtered at 85% coverage and [file 13073_2024_1360_MOESM1_ESM.docx]

**Additional file 1**


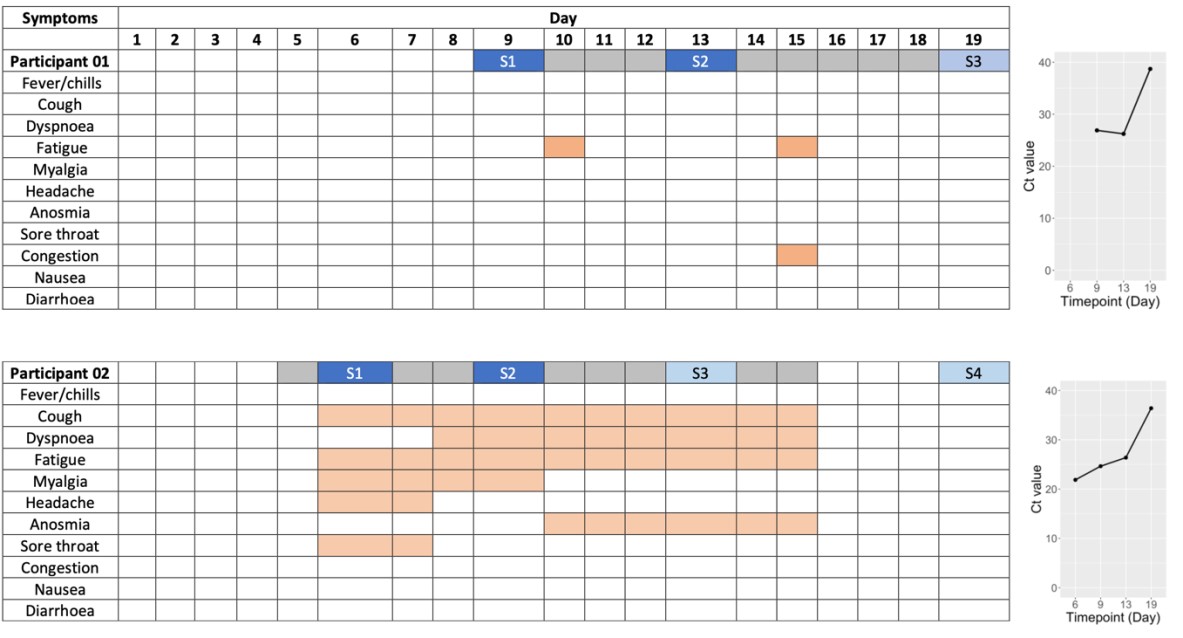


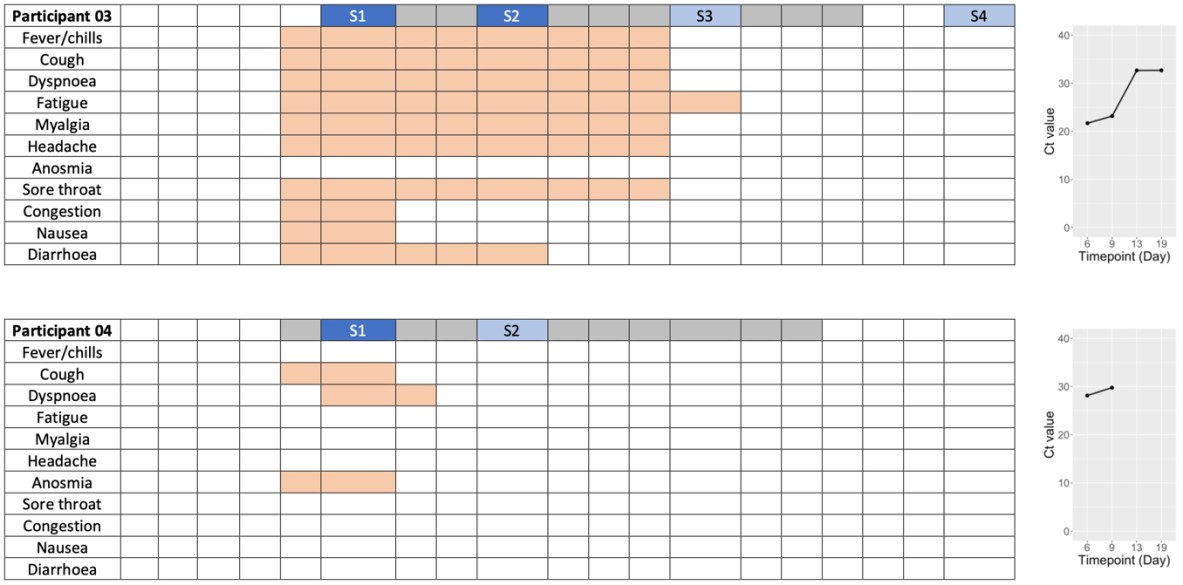


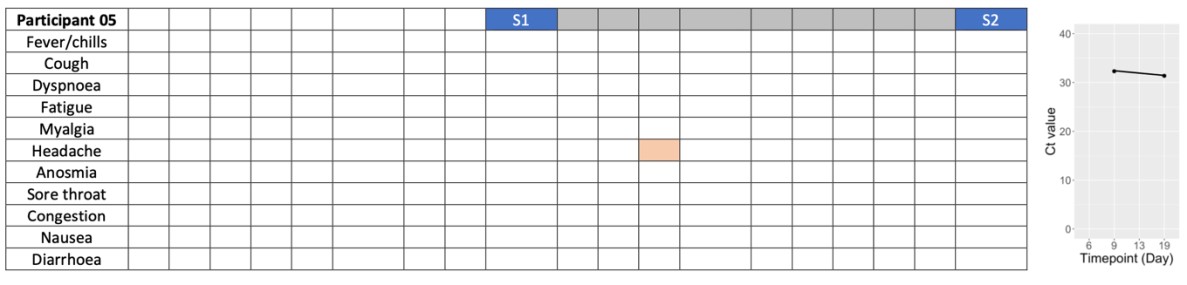


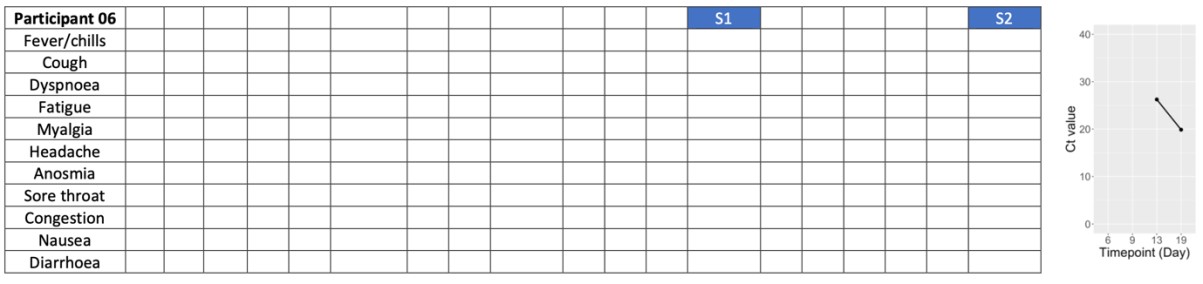

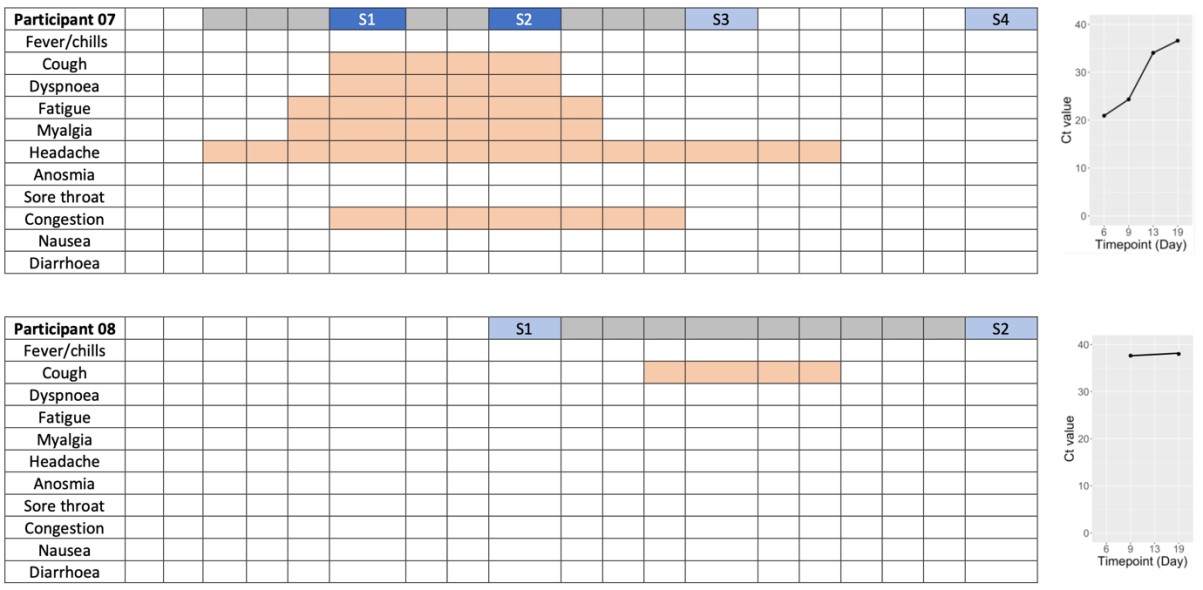

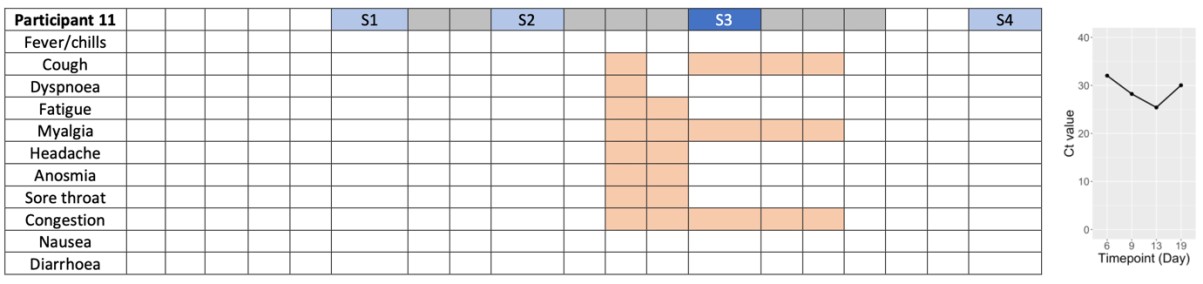

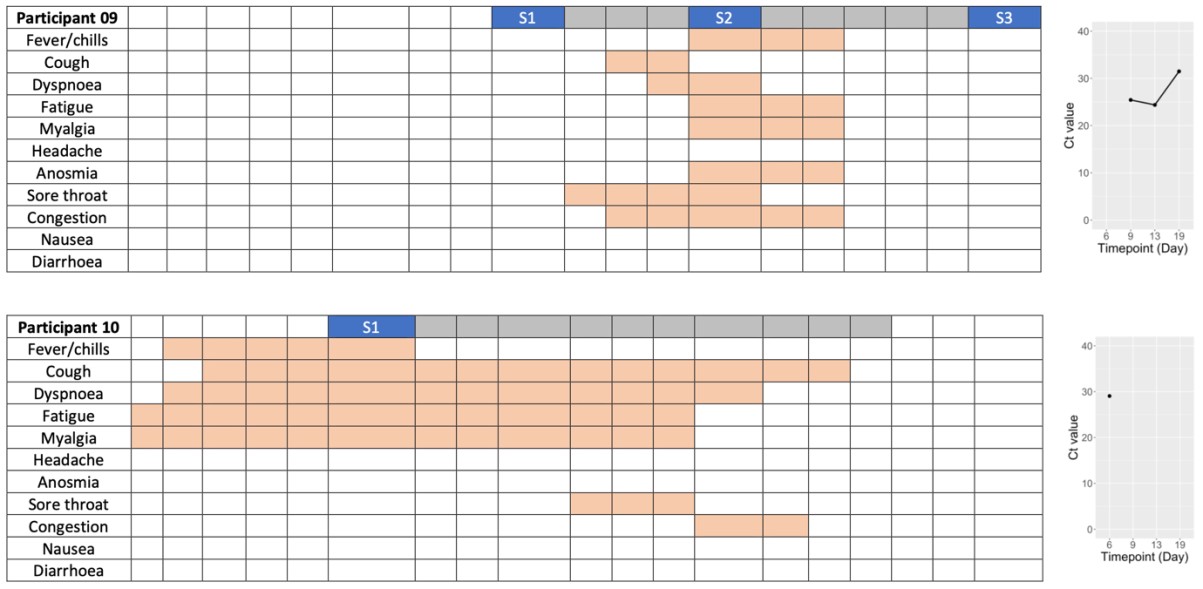

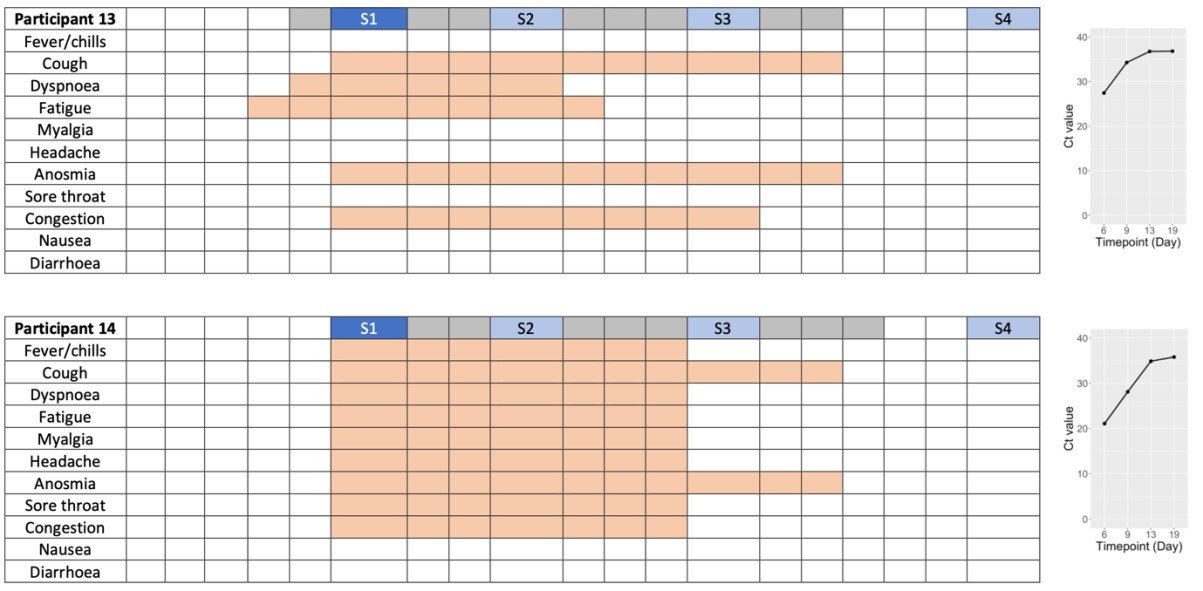

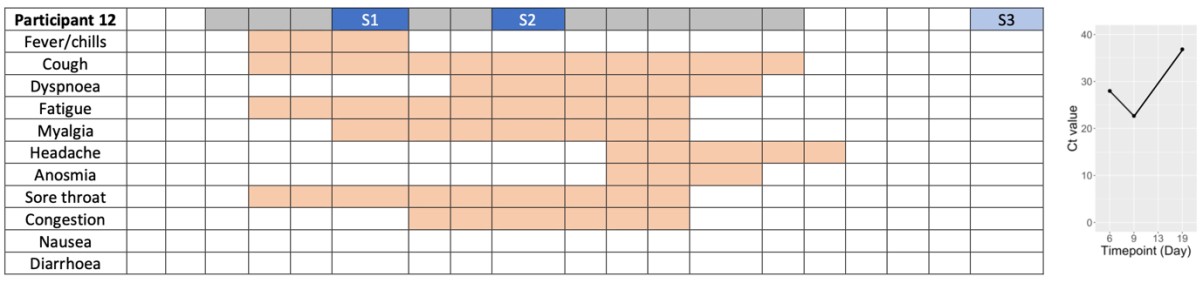


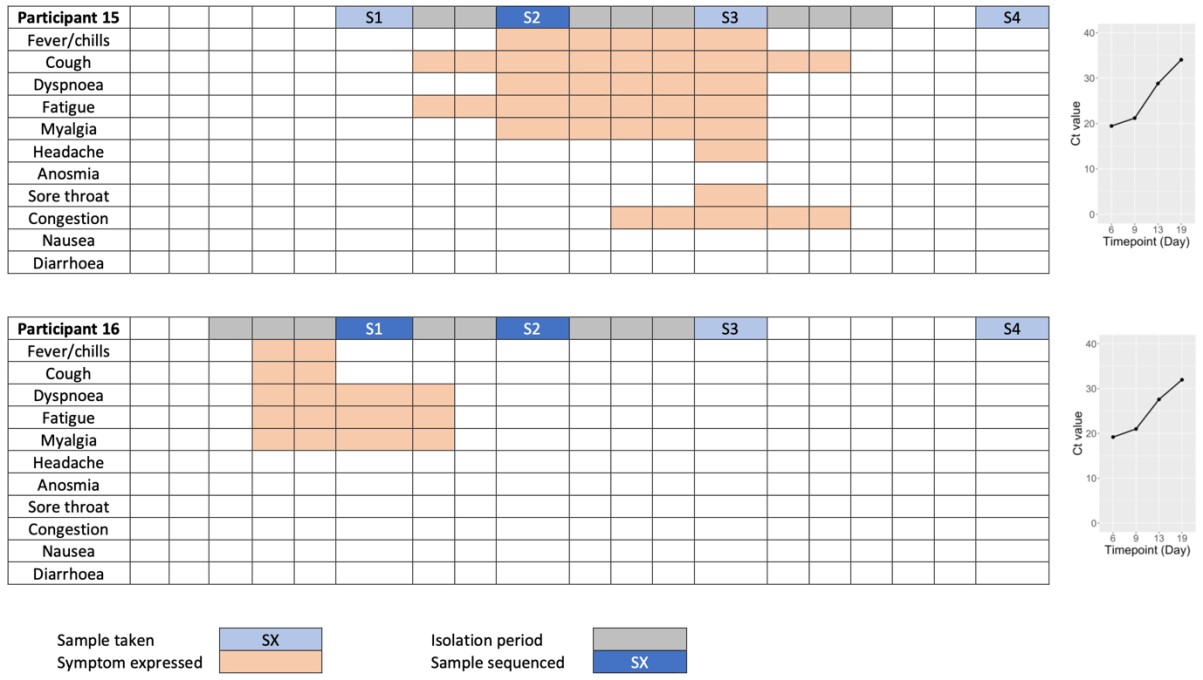


**Figure S1. Participant symptomatology during the study with corresponding Ct values from positive E-gene RT-qPCR results.** Symptomatology reported over the course of the study from day 0 (the timepoint of the super-spreading event) showing when the samples were taken (light blue shading) and their corresponding Ct values from a positive E-gene RT-qPCR test (on the right-hand graph). Symptoms reported (shown down the left-hand side) are shown in light orange, isolation period is shown in grey and samples that were sequenced subsequent to collection are shown in dark blue.


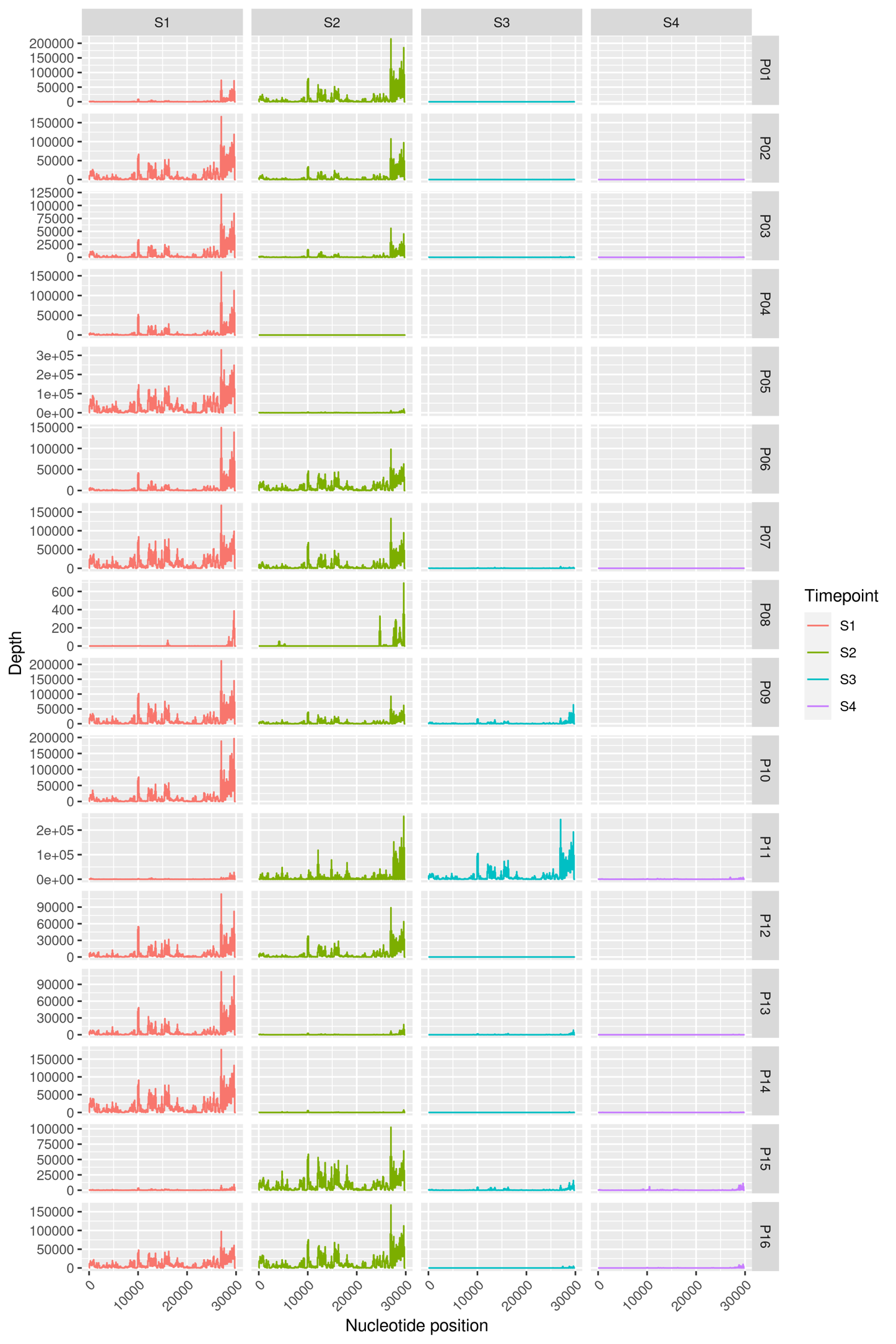


**Figure S2. Genome sequencing coverage and depth plots for all SARS-CoV-2 samples collected from participants (timepoints are shown as S1-4 where S=sample as timepoints were different between participants), including those with <85% coverage which were not subsequently analysed.** Depth was calculated using SAMtools to give coverage across the length of the genome and depth per nucleotide position. Participants (P) are down the right-hand side, with timepoints shown across the top and genome depth using free axes on the y-axis.


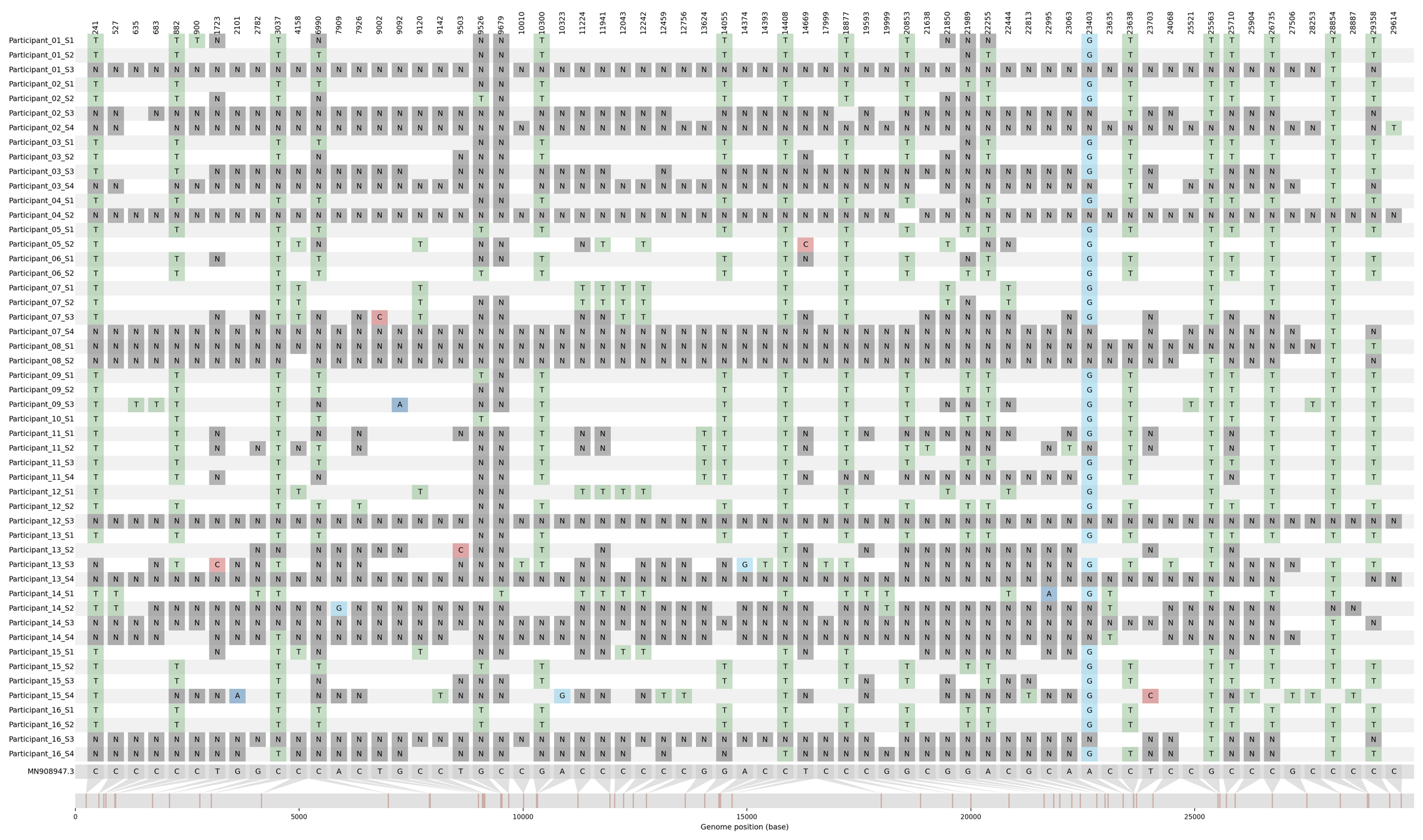


**Figure S3. Nucleotide mutations across the genomes of all participant samples compared to the reference genome (MN908947.3).** Diagram of all the nucleotide mutations where sufficient coverage was obtained (20X at that position), insufficient coverage is reported as N at that position generated via snipit.


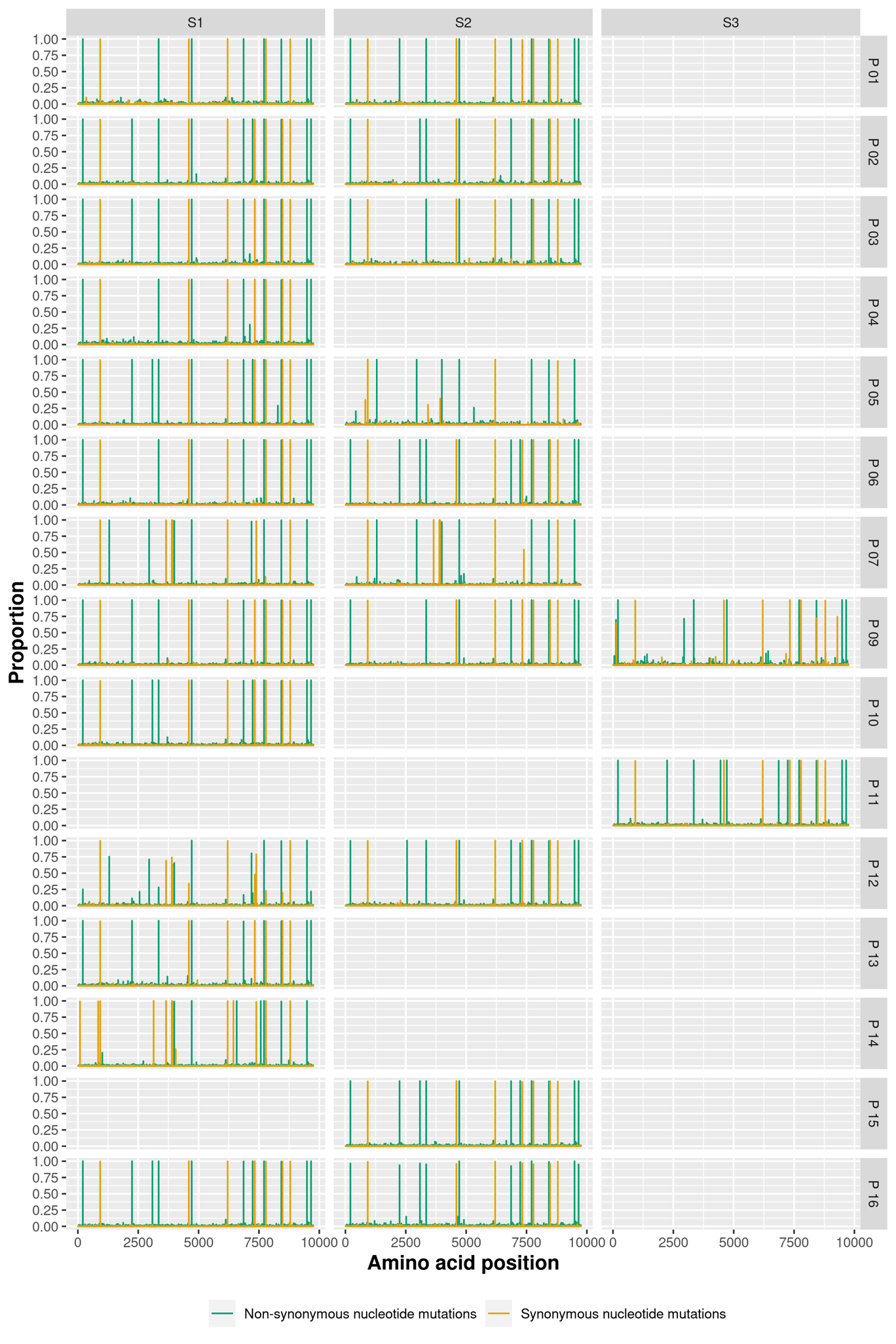


**Figure S4. Proportion of non-synonymous (blue) and synonymous (orange) amino acid variation across the genomes of the different participant samples (S1, S2, S3) with >85% coverage, compared to the reference genome.** Dominant amino acid substitutions were observed at a proportion of >0.5, with many lineage defining mutations near a proportion of 1, and minor variants can be seen at a proportion of generally <0.5 and generally at a low level across the genome. Coverage filtered at 20X.

**Table S1. Coverage of samples sequenced when filtered at 85% coverage and then 20X and 10X depth and base quality scores of reads post trimming.** R1 and R2 represent Illumina paired-end reads read 1 (R1) and read 2 (R2).
